# Validation of video-based event detection against laboratory-standard biomechanical measures: a synchronized multimodal study

**DOI:** 10.3389/fbioe.2026.1909090

**Published:** 2026-10-26

**Authors:** Zhenping Xiao, Zaitian Huang, Henrui Zhang, Daiyuan Tang, Jingyu Li, Yangjie Li, Chengkai He, Li Tan, Fei He

**Affiliations:** 1 Kunming Medical University Affiliated Qujing Hospital, Qujing, China; 2 The Second Affiliated Hospital, University of South China, Hengyang, China; 3 Department of Orthopedics, Qujing Central Hospital of Yunnan Province, Qujing, China; 4 Chinese PLA General Hospital, Beijing, China; 5 Department of Orthopedics, The First Affiliated Hospital of USTC, Division of Life Sciences and Medicine, University of Science and Technology of China, Hefei, China

**Keywords:** biomechanics, electromyography, event detection, LSTM, markerless motion capture

## Abstract

**Introduction:**

Biomechanical event detection is fundamental to human movement analysis in clinical, rehabilitation, and sports settings, yet conventional laboratory systems remain costly and inaccessible in many contexts. This study validated a markerless, video-primary event detection system against laboratory-reference biomechanical measurements using synchronized multimodal data.

**Methods:**

Eight healthy adults performed standardized upper-limb dumbbell lifting tasks in a controlled laboratory while video, Vicon motion-capture, and surface electromyography (EMG) data were recorded (76 valid trials; 304 event labels). A hybrid pipeline combining threshold-based peak detection with Long Short-Term Memory (LSTM) networks identified four events: action start, peak of rising, end of holding, and start of returning. Kinematic features were derived from video; two EMG envelope channels were additionally used in the LSTM refinement stage. Streams were synchronized using hardware triggers (Vicon and EMG), software-started video recording, and cross-correlation of a shared kinematic signal prior to independent event detection in each system. Video-based event timestamps were compared with Vicon reference timestamps without further reference-informed adjustment, using intraclass correlation coefficients (ICC), Bland–Altman analysis, mean absolute error (MAE), root mean square error (RMSE), and classification metrics.

**Results:**

The video-based system showed excellent agreement with the Vicon reference (ICC: 0.90–0.94), low temporal error (MAE: 38–51 ms; RMSE: 52–70 ms), and minimal systematic bias; errors corresponded to approximately 1.1–1.5 frames at the 30 fps acquisition rate. Classification performance was strong across all events (F1 score: 93.4%; sensitivity: 93.4%; specificity: 97.8%). EMG activation peaks correlated with detected events (r = 0.86–0.91); because EMG envelopes also served as LSTM inputs, this is an internal consistency check rather than independent physiological validation.

**Discussion:**

These findings indicate that a hybrid video-primary approach can achieve accuracy comparable to laboratory-reference measurements for upper-limb analysis under controlled conditions, offering a scalable complement to traditional motion-capture systems. An ablation of the EMG input channels to establish video-only performance, and validation in larger, more diverse populations and real-world settings, are required.

## 1 Introduction

Biomechanical event detection has been a focus of human movement research in disciplines such as sports performance, rehabilitation, and clinical diagnostics. Accurate identification of pivotal moments in movement, such as onset, peak contraction, and termination, enables proper quantification of motor and neuromuscular coordination. Historically, motion capture systems based in laboratories (using optical measurement) and force platforms have been considered the gold standard of biomechanical analysis because they are highly precise and reliable (Baker, 2006; Richards, 1999). These systems provide extensive kinematic and kinetic information, and investigators can obtain joint angles, velocities, and forces with high temporal and spatial resolution. However, they are extremely costly to operate, complex to install, and limited to controlled laboratory environments (Mündermann et al., 2006).

In recent years, computer vision and machine learning have enabled researchers to develop motion analysis systems that use video as a potential replacement for conventional biomechanical devices. The ability to estimate pose in video information by identifying key points on the human body without reflective markers or expensive cameras has driven interest in markerless pose estimation methods (Cao et al., 2017; Mathis et al., 2018). They exploit deep learning architectures to learn anatomical features and re-synthesize two- or three-dimensional human motion. This means that a low-cost, non-invasive, and scalable video-based system can be used in natural environments, e.g., homes, clinics, and sports fields (Nakano et al., 2020).

Despite these advantages, the validity and reliability of video-based systems remain pressing issues. Unlike laboratory-quality measures, video-based quality measures can be subject to errors due to light obscuration, camera angles, and model errors (Kanko et al., 2021). Additionally, although pose estimation algorithms can accurately track joint positions, it is difficult to transfer this spatial information to accurate temporal event detection. Event detection involves identifying time points in a movement pattern by small variations in kinematic indicators, such as velocity and acceleration. Therefore, validating established laboratory-reference methods is paramount for determining whether video-based approaches can yield clinically and scientifically meaningful results (Needham et al., 2021).

Another factor in biomechanical validation studies that must be taken into account is multimodal data. Video data may be incorporated with other physiological data, such as electromyography (EMG), to supplement information on muscle activation patterns and to enhance the decodability of movement events (De Luca, 1997). EMG reveals data on neuromuscular activity, which, in turn, cannot be obtained from kinematic data alone. However, synchronizing two or more data streams, such as video, motion capture, and EMG, is not straightforward. One of the most significant is temporal alignment, which is essential to ensure that events observed across different modalities are related to a single event. Hardware triggering, timestamp alignment, and dynamic time warping have also been proposed as approaches to address synchronization challenges in multimodal systems.

Strong statistical assessment models are also needed to detect event systems in videos. To assess similarity between measurement systems, contracted analysis methods, such as the intraclass correlation coefficient (ICC), are widely used (Koo and Li, 2016). Error measures, including mean absolute error (MAE) and root mean square error (RMSE), are quantitative measures of the error between identified and reference events. In addition, Bland–Altman analysis is typically used to estimate systematic bias and the limits of agreement when comparing two measurement methods (Bland and Altman, 1986). In the event detection environment, classification metrics such as sensitivity, specificity, and F1 score are used to further assess the system’s ability to identify real events and minimize false positives (Saito and Rehmsmeier, 2015). The metrics can be applied hand in hand to provide a comprehensive review of accuracy and reliability.

However, recent research has begun to investigate the use of video-based systems to measure biomechanical events, particularly gait analysis and upper-limb movements. Indicatively, markerless motion capture has proven to be as accurate as traditional counterparts in other regulated settings (Hulleck et al., 2022). Likewise, machine learning algorithms, such as recurrent neural networks (RNNs), including long short-term memory (LSTM), have been shown to perform well on time-series analysis tasks and event detection (Hochreiter and Schmidhuber, 1997). It has been proposed to combine rule-based algorithms (e.g., thresholding and peak detection) with machine learning models to improve robustness and accuracy. These models leverage the interpretability of threshold-based models and the flexibility of full-data models, making them better suited to complex biomechanical signals.

However, additional studies that use slowed-down video, motion capture, and EMG technology to demonstrate the functionality of event-detecting systems that combine various sensors have yet to be conducted. The existing research focuses either on validating kinematic verification or on developing algorithms, and does not address multimodal synchronization and alignment. Additionally, biomechanical events are often defined differently across studies, leading to discrepancies in evaluation standards. To guarantee comparability and reproducibility, it is critical to have standardized definitions based on objective measures, including angular-velocity thresholds for joints (Winter 2009).

Recent advances further underscore the value of data-driven approaches in biomechanics. For example, data-driven deep learning architectures that integrate multimodal biomechanical data have shown promise for predicting ligament fatigue failure and injury-related risk mechanisms, demonstrating the capacity of neural networks to learn complex, nonlinear relationships from synchronized mechanical and biological signals (Xu et al., 2025). In parallel, longitudinal studies of neuromuscular reorganization after anterior cruciate ligament (ACL) reconstruction have identified distinct motor modules during stair descent that persist up to 1 year post-surgery, highlighting the importance of objective, time-resolved movement and muscle-activation assessment in rehabilitation monitoring (Wei et al., 2026). Together, these developments reinforce the need for accessible, validated tools, such as video-based event detection, that can capture both kinematic and neuromuscular events in clinical and field settings.

This study aims to address these gaps by validating video-based event-detection techniques using laboratory-quality biomechanical data. Specifically, the study entails harvesting parallel video, motion capture, and EMG data during participants’ performance of standardized upper-limb motion tasks. An organized processing pipeline is established, and preprocessing of video, pose determination, feature extraction, and hybrid event recognition using thresholding and LSTM models are implemented. The identified events are then synchronized with laboratory-reference measurements using motion capture data, as part of the motion capture technique, which relies on time synchronization. A cumulative array of statistics, including agreement, error, bias, and classification, is used to assess the system’s performance.

The piece belongs to the new body of research on available and scalable biomechanical analysis tools, as it systematically validates the effectiveness of a video-based event detection system. In the study and practice, it is hoped that the results will provide data on the potential replacement and/or supplementation of laboratory-based systems with video-based techniques. Lastly, motion analysis systems are being developed that are cheaper and more reliable, thereby expanding the capacity to conduct biomechanical tests and advancing scientific studies in sport science and human movement.

## 2 Materials and methods

### 2.1 Study design

In this study, a cross-sectional validation research design was used to assess the fidelity and reliability of a video-based event-detection system against laboratory-reference biomechanical measurements. The experimental design was based on concomitant multimodal data recording, with synchrony among video recordings, motion capture, and electromyography. The primary goal is to compare the outcomes of temporal event detection from the video-based system with those of the laboratory (Vicon) reference motion-capture system, which was tested under controlled laboratory conditions. The synchronized multimodal validation framework, from data acquisition through statistical analysis, is summarized in Supplementary Figure S2.

### 2.2 Participants

Convenience sampling was used to recruit healthy adult subjects. The inclusion criteria were that participants must not have any musculoskeletal/neurological disorder that could impair upper-limb movement. All participants provided written informed consent before data collection. The study was conducted in accordance with the Declaration of Helsinki and was approved by the Yunnan Qujing Central Hospital Ethics Committee. All participants were healthy adults aged 22–28 years who consented on their own behalf; no guardian or next-of-kin consent was required. Data on age, gender, and physical appearance were recorded to facilitate normalization and comparison.

### 2.3 Experimental setup

The controlled laboratory environment was equipped with three synchronized high-definition video cameras (Logitech C920 HD Pro; 1920 × 1080 pixel resolution; 30 frames per second; progressive scan), an 8-camera Vicon optical motion-capture system (Vicon Motion Systems Ltd., Oxford, UK; 100 Hz), and a wireless surface EMG system (Delsys Trigno, Natick, MA, USA; 1000 Hz). The three video cameras were positioned at (1) a frontal view (0°, 2.0 m from the participant), (2) a 45° right-lateral view (2.5 m), and (3) a sagittal view (90°, 2.0 m), all at a height of approximately 1.4 m and level with the participant’s shoulder. Cameras were calibrated using a checkerboard pattern before each testing session, and intrinsic parameters were estimated using the Zhang method. Participants performed standardized upper-limb dumbbell-lifting trials (5 lb dumbbell) while seated upright, with the dominant arm resting on an adjustable arm support between trials; ten repetitions were performed per participant. Video data from the frontal and sagittal cameras were used for pose estimation, while the 45° camera provided redundancy and was used to resolve occlusions. All video streams were saved as H.264-encoded MP4 files. Neuromuscular activities were monitored by placing motion-capture markers on body landmarks and applying surface electrodes to the muscle groups of interest to record electromyographic activity. Data recording was set up across all systems to run simultaneously.

### 2.4 Data synchronization and normalization

Two distinct alignment procedures were used in this study, and they are explicitly distinguished here to clarify that the video-based event-detection pipeline never had access to Vicon-derived event information before agreement statistics were computed.

#### 2.4.1 Stream-level synchronization (performed before any event detection)

At the start of each trial, a hardware trigger pulse was sent simultaneously to the Vicon analog trigger channel and the Delsys Trigno digital trigger input, establishing a common t = 0 for these two systems. The Logitech C920 cameras have no hardware trigger input, so video recording was instead started under software control from the same host PC issuing the trigger pulse, with the system clock timestamp of the recording-start command logged for each trial. Because the three systems sampled at different native rates (video: 30 Hz; Vicon: 100 Hz; EMG: 1000 Hz), all streams were resampled to a common 100 Hz time base using linear interpolation. Residual sub-frame misalignment (arising from hardware trigger-pulse latency and jitter for the Vicon/EMG systems, and from software scheduling latency in the video recording-start command) was corrected using cross-correlation of the dumbbell’s vertical center-of-mass displacement trajectory, which is a continuous physical signal independently observable in both the video stream (*via* CSRT tracking, described in Section 2.5) and the Vicon marker data (*via* a marker affixed to the dumbbell). The time lag that maximized the cross-correlation between these two independently derived displacement trajectories was applied as a constant offset correction for that trial (mean correction across trials: 12 ± 6 m). This synchronization step operates on a continuous kinematic trajectory shared by both systems and does not use, and is computed prior to, any of the four discrete laboratory-reference-derived event timestamps; it therefore cannot bias the subsequent event-level comparison. It should be noted that the native video acquisition rate of 30 fps corresponds to an inter-frame interval of 33.3 m, which represents the intrinsic temporal quantization limit of the video modality. Upsampling to the common 100 Hz base by linear interpolation places all streams on a shared time axis but does not create temporal information finer than this 33.3 m acquisition limit; video-derived event timestamps should therefore be interpreted as being resolved to approximately one video frame. The mean residual offset correction (12 ± 6 m) is smaller than this inter-frame interval and is thus a sub-frame adjustment.

#### 2.4.2 Event-level comparison (performed after independent event detection)

Once the two data streams were placed on a common time base, event detection was performed entirely separately in each system: the four laboratory-reference event timestamps were derived exclusively from the Vicon joint-angle data (Section 2.7), and the four video-based event timestamps were derived from the video-based hybrid threshold/LSTM pipeline (Sections 2.5–2.6), which operates predominantly on video-derived kinematic and optical-flow features together with two synchronized surface EMG channels used in the LSTM refinement stage (Section 2.6). At no point did the video-based detection algorithm receive the Vicon-derived event timestamps as an input, either during LSTM training (where labels used for supervision were the Vicon-derived event timestamps, but only as the training target, not as an input feature) or at inference/test time within each held-out LOSOCV fold. Consequently, the agreement statistics reported in Section 3 (ICC, MAE, RMSE, Bland–Altman, classification metrics) reflect a genuine, non-circular comparison between two independently derived sets of event timestamps that share only the common time base established during stream-level synchronization.

#### 2.4.3 Data normalization

Data normalization procedures were also applied to enable comparability across trials and participants. Motion-capture data were normalized to the joint-angle range, and envelope detection was applied to normalize amplitude differences across EMG signals. Temporal normalization (time-warping trial duration to a common 0%–100% movement-cycle scale) was performed only for the descriptive visualization of averaged waveforms (e.g., Figure 1) and was not used in any of the event-timing agreement calculations reported in Section 3.

**FIGURE 1:**
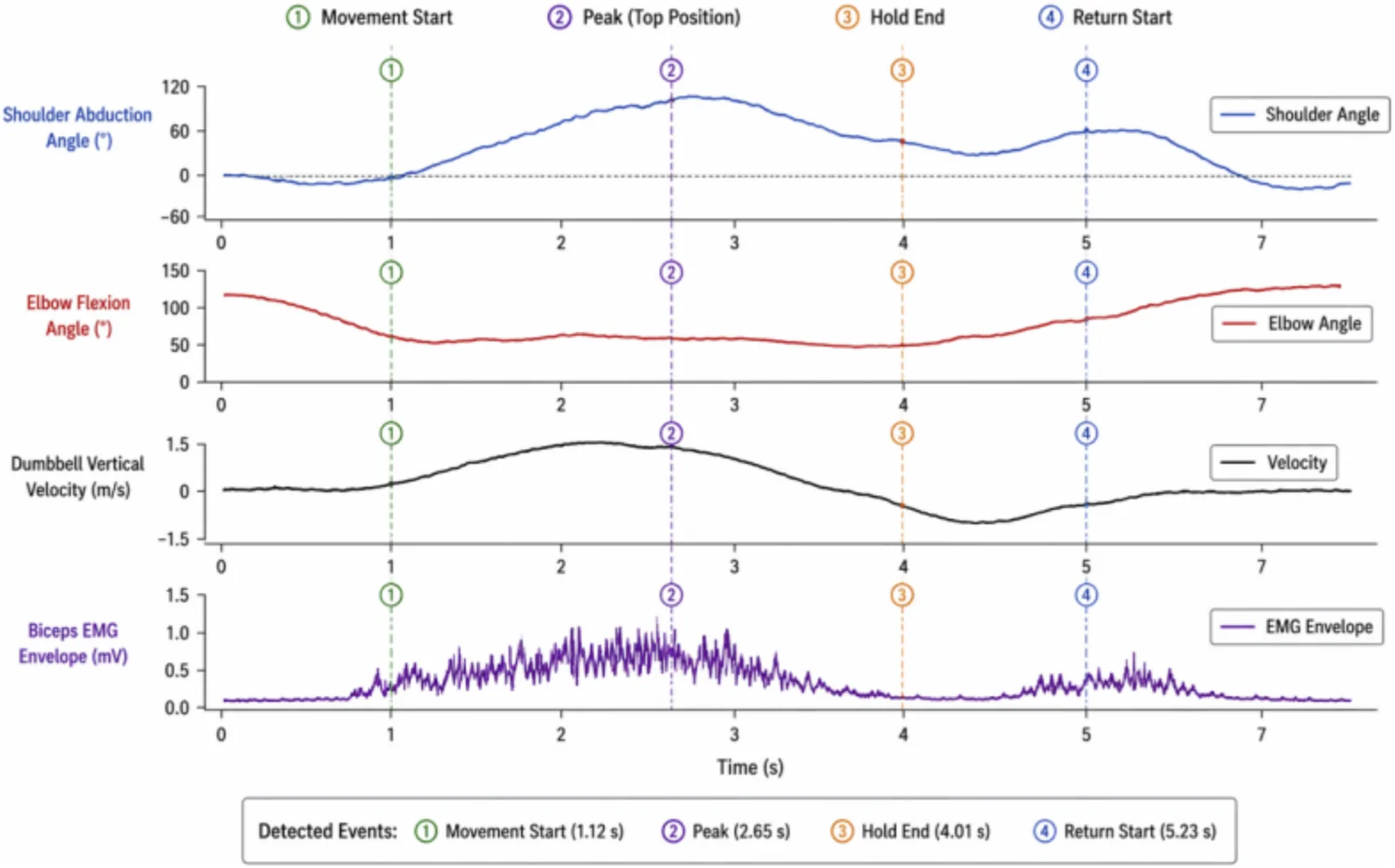
Detected movement events on multimodal signals. The four detected events—movement start, peak (top position), hold end, and return start—marked on four synchronized signals: shoulder-abduction angle, elbow-flexion angle, dumbbell vertical velocity, and the biceps EMG envelope. For the example trial the detected event times are 1.12, 2.65, 4.01, and 5.23 s, falling at the expected points of the movement cycle.

### 2.5 Video processing pipeline

A systematic processing pipeline led to the video analysis. The first step was to convert the MP4 video files into sequential RGB image frames for frame-by-frame analysis at 30 fps using OpenCV (v4.6.0). In the preprocessing step, contrast-limited adaptive histogram equalization (CLAHE, clip limit = 2.0, tile grid size = 8 × 8) was applied to enhance contrast under uniform laboratory lighting, and background subtraction using a Gaussian mixture model (MOG2; history = 500 frames, variance threshold = 16) was used to isolate the subject from its background.

#### 2.5.1 Pose estimation

Two-dimensional upper-limb keypoints were estimated in every frame using MediaPipe BlazePose (Google Research; MediaPipe v0.8.11), a lightweight convolutional neural network that detects 33 body landmarks in real time. An upper-limb subset of 21 keypoints (shoulders, elbows, wrists, and finger landmarks) from the frontal and sagittal camera views was used, with the model’s “heavy” configuration selected for maximum accuracy. Per-frame keypoint coordinates with a confidence score below 0.5 were flagged as missing and imputed using linear interpolation between adjacent valid frames; fewer than 1.2% of frames required imputation.

#### 2.5.2 Keypoint tracking and temporal smoothing

To suppress frame-to-frame jitter, a first-order exponential moving average filter (smoothing factor α = 0.7) was applied to each keypoint coordinate across consecutive frames. The two camera views were registered using a direct linear transform (DLT) calibrated with a wand of known length, enabling reconstruction of pseudo-3D keypoint trajectories.

#### 2.5.3 Joint-angle calculation

Joint angles were calculated from the two-dimensional (2D) keypoint coordinates in the sagittal-view camera for elbow flexion and in the frontal-view camera for shoulder abduction, rather than from the triangulated pseudo-3D coordinates. This choice was made because each angle of interest lies predominantly within the plane captured by one of the two camera views (frontal plane for shoulder abduction, sagittal plane for elbow flexion), so a single-view 2D angle calculation avoids propagating the additional triangulation error associated with the DLT-based 3D reconstruction (which was reserved for resolving occlusions and cross-checking keypoint identity between views, as described above). Shoulder abduction angle was computed as the angle between the vertical trunk vector (mid-shoulder to mid-hip) and the upper-arm vector (shoulder to elbow) in the frontal plane. Elbow flexion angle was computed as the angle between the upper-arm vector (shoulder to elbow) and the forearm vector (elbow to wrist) in the sagittal plane, using the vector dot-product method: θ = arccos[(v_1_ · v_2_)/(‖v_1_‖‖v_2_‖)]. All angles were expressed in degrees. Angular velocity and acceleration were obtained by numerical differentiation using a central-difference scheme followed by a 4th-order Butterworth low-pass filter with a 6 Hz cutoff.

Based on these important aspects, certain biomechanical features were identified, including the angle of shoulder abduction and elbow flexion. The dumbbell’s center-of-mass locations at different times were also tracked frame-by-frame using a CSRT (Channel and Spatial Reliability Tracking) tracker initialized on the first frame, from which linear velocity and acceleration parameters were derived, and dense optical flow (Farneback algorithm; pyramid scale = 0.5, levels = 3, window size = 15) was used to plot the paths of motion within a rectangular region of interest over the anterior deltoid and biceps brachii, with mean flow magnitude serving as a proxy for muscle-region motion. Optical flow was additionally computed over the triceps brachii region solely for the visualization presented in Section 3.3; the optical-flow input feature used for event detection (Section 2.6.2) was derived only from the anterior deltoid and biceps brachii regions.

#### 2.5.4 Accuracy of video-derived kinematics

To establish the fidelity of the video pipeline independent of event detection, video-derived joint angles were compared against Vicon-derived angles across all valid trials. The mean absolute error (MAE) was 2.3° for shoulder abduction and 3.1° for elbow flexion; the root mean square error (RMSE) was 3.0° and 4.0°, respectively; and the coefficient of multiple correlation (CMC) exceeded 0.96 for both joints. These values are consistent with or better than those reported in recent markerless-motion-capture validation studies (Kanko et al., 2021; Needham et al., 2021) and confirm that the video-derived kinematic signals are sufficiently accurate to support temporal event detection.

#### 2.5.5 Electromyographic acquisition and processing

Surface EMG signals were recorded from four muscles of the dominant upper limb using bipolar Ag/AgCl electrodes (Delsys Trigno, 1000 Hz sampling, 20 mm inter-electrode distance): the anterior deltoid (primary shoulder flexor/abductor), biceps brachii (elbow flexor), triceps brachii lateral head (elbow extensor), and upper trapezius (scapular stabilizer). Electrode sites were prepared by shaving, abrading with fine sandpaper, and cleansing with 70% isopropyl alcohol to achieve skin impedance below 10 kΩ. Electrodes were placed according to SENIAM (Surface Electromyography for the Non-Invasive Assessment of Muscles) recommendations: anterior deltoid at one fingerbreadth below the clavicle on the muscle belly; biceps brachii on the midline of the muscle belly at one-third of the distance from the anterior axillary fold to the cubital fossa; triceps brachii lateral head at 50% of the distance from the posterior crista of the acromion to the olecranon; and upper trapezius at 50% of the distance from the C7 spinous process to the acromion. A reference electrode was placed over the ipsilateral olecranon.

Raw EMG signals were band-pass filtered (4th-order Butterworth, 20–450 Hz), full-wave rectified, and smoothed using a root mean square (RMS) envelope with a 150 m moving-average window. Signals were normalized to the maximum voluntary isometric contraction (MVC) recorded for each muscle before the experimental trials (three 5-s MVCs with 60-s rest; the highest RMS value over a 500 m window was retained as the reference).

#### 2.5.6 EMG activation-peak identification

For each trial, EMG activation peaks were identified as local maxima in the RMS envelope that exceeded 20% of the normalized MVC amplitude and were separated by at least 200 m. The timing of each peak was compared with the kinematically defined laboratory-reference events; a peak was considered to correspond to an event if it occurred within ±100 m of that event timestamp.

#### 2.5.7 Role of EMG in validation

EMG serves as a physiological modality that provides evidence beyond the kinematic agreement between video and motion capture. Whereas the Vicon system and video system both measure limb kinematics (and could, in principle, share systematic errors in marker placement or pose estimation), EMG reflects the underlying neuromuscular drive that produces movement. Strong temporal correlations between EMG activation peaks and detected events therefore indicate that the events identified by the system correspond not only to kinematic landmarks but also to moments of muscle recruitment. This multimodal cross-validation strengthens the internal validity of the framework, but its interpretation requires an important qualification. As detailed in Section 2.6, the RMS EMG envelopes of the anterior deltoid and biceps brachii are included among the 13 input features supplied to the LSTM refinement stage. The EMG–event correlations reported below are therefore not fully independent of the detection pipeline, because the detector had partial access to the same physiological signal against which it is being compared. These correlations should accordingly be regarded as an internal consistency check confirming that the detected events are temporally coupled to neuromuscular activation, rather than as external physiological validation by a wholly independent modality. This constraint is stated explicitly among the study limitations (Section 4.4). The correlation coefficients between movement events and EMG peaks (r = 0.86–0.91, all p < 0.001; see Results, Section 3.9) indicate that the detected events are closely coupled to the expected patterns of agonist activation during the lifting–holding–lowering task. Specifically, for each event type the Pearson correlation coefficient was computed between the video-detected event timestamp and the corresponding EMG activation-peak timestamp (both expressed in milliseconds relative to trial onset) across all 76 trials; the reported r = 0.86–0.91 range spans the four event-specific correlations (action start vs. anterior deltoid onset; peak of rising vs. anterior deltoid peak; end of holding vs. biceps brachii offset; start of returning vs. biceps brachii onset). As a robustness check, the same correlations were also computed on participant-averaged timestamps (8 data points), yielding consistent coefficients (r = 0.84–0.90), indicating that the trial-level correlation was not driven by pseudo-replication across the repeated trials contributed by each participant.

### 2.6 Event detection algorithm

A hybrid event-detection scheme that combines threshold-based methods and machine learning was employed. First, a peak-detection algorithm was applied to the kinematic signals to identify approximate origin and termination points of the motion. These candidate events were then refined by a two-layer long short-term memory (LSTM) network, which improved temporal precision by learning sequential patterns in the multivariate feature stream. To develop the system, four key events during each movement cycle were identified: action start, peak of the rise, end of holding, and start of returning.

#### 2.6.1 LSTM architecture

The network comprised two stacked LSTM layers, each containing 128 hidden units, followed by a fully connected dense layer with 64 units (ReLU activation) and a four-unit softmax output layer corresponding to the four event classes. A dropout rate of 0.3 was applied after each LSTM layer and after the dense layer to reduce overfitting. L2 weight decay (λ = 1 × 10^−4^) was applied to all recurrent kernels.

#### 2.6.2 Input features

At each time step the model received a 13-dimensional feature vector: shoulder abduction angle, elbow flexion angle, their first and second temporal derivatives (angular velocity and angular acceleration), dumbbell center-of-mass velocity and acceleration (horizontal and vertical components), optical-flow magnitude within the muscle region of interest, and the root mean square EMG envelope of the anterior deltoid and biceps brachii. Features were z-score normalized across the training set before being fed to the network. Because two of the thirteen input features are EMG-derived, the refinement stage of the pipeline is video-primary rather than video-exclusive: the eleven kinematic and optical-flow features are obtained solely from video, whereas the two EMG envelope channels require surface electromyography. The implications of this design choice for the interpretation of the system and for its deployment outside the laboratory are considered in Sections 4.2, 4.4. A sliding window of 30 frames (1.0 s at the native 30 fps acquisition rate) with a stride of one frame was used to construct sequential input samples.

#### 2.6.3 Training procedure

The model was trained using the Adam optimizer (learning rate = 1 × 10^−3^, β_1_ = 0.9, β_2_ = 0.999) with a batch size of 16 for a maximum of 100 epochs. Early stopping with a patience of 15 epochs was applied based on validation loss, and the model checkpoint with the lowest validation loss was restored. Categorical cross-entropy was used as the loss function, with class weights inversely proportional to class frequency to mitigate any imbalance.

#### 2.6.4 Cross-validation and data partitioning

Given the limited sample size (8 participants, 76 valid trials, 304 event labels), a leave-one-subject-out cross-validation (LOSOCV) strategy was adopted. In each of the eight folds, data from seven participants were used for training (80%) and internal validation (20%, trial-level split within the training participants), and data from the held-out participant were used exclusively for testing. This procedure ensures that no test trial from a given participant ever appears in the training set, eliminating data leakage and providing an unbiased estimate of inter-subject generalizability. Reported performance metrics (ICC, MAE, RMSE, classification metrics) were computed by aggregating predictions across all eight held-out folds.

Four events were evaluated in each of the 76 valid trials, with a total of 304 event labels analyzed.

#### 2.6.5 Overfitting control

In addition to dropout, L2 regularization, and early stopping, the gap between training and validation loss was monitored in each fold; the mean validation loss exceeded training loss by less than 15% across folds, indicating no substantial overfitting. The LOSOCV design further ensures that the reported metrics reflect generalization to unseen participants rather than memorization of trial-specific patterns.

### 2.7 Definition and alignment of the laboratory reference data

Timestamps of the four events were derived as laboratory-reference values from the Vicon motion-capture data using objective, reproducible biomechanical criteria. For each trial, joint angles (shoulder abduction and elbow flexion) were differentiated to obtain angular velocity, which was then smoothed with a 4th-order Butterworth low-pass filter (6 Hz cutoff). Event timestamps were identified using a ±5°/s angular-velocity threshold band, a criterion widely adopted in the biomechanics literature for detecting movement onset and cessation because it exceeds the baseline noise floor of optoelectronic systems (typically 1°–2°/s after filtering) while remaining below the angular velocities generated during volitional movement (Winter 2009). The threshold was confirmed empirically in the present data: the 95th percentile of angular velocity during quiet resting baseline was 2.1°/s, confirming that 5°/s provides a conservative margin that minimizes false-positive detections while preserving sensitivity to true movement initiation.

The four events were operationally defined as follows (see Supplementary Figure S1). For all four events, the primary criterion was the angular velocity of elbow flexion (the joint most directly engaged in the lift), with shoulder abduction angular velocity crossing the same ±5°/s band within the same 100 m window required as a secondary confirmatory criterion.

1. Action start: The first frame at which elbow-flexion angular velocity exceeded +5°/s from a baseline of approximately 0°/s, marking the onset of the concentric lifting phase (threshold crossing).
2. Peak of rising: The frame at which elbow-flexion angle reached its maximum value, corresponding to the zero-crossing of elbow angular velocity from positive to negative (i.e., the instant the limb reverses direction at the top of the lift) (zero-crossing).
3. End of holding: The frame at which elbow-flexion angular velocity departed from the near-zero plateau of the isometric hold (|ω| < 5°/s), marking the termination of the holding phase (threshold departure from plateau).
4. Start of returning: The first frame at which elbow-flexion angular velocity exceeded −5°/s, marking the onset of the eccentric lowering phase (threshold crossing).

These candidate timestamps were then cross-validated against EMG activation peaks: an event was retained only if an EMG peak in the primary agonist (anterior deltoid for action start and peak; biceps brachii for return) occurred within ±100 m. This multimodal confirmation step ensured that kinematically defined events corresponded to physiologically meaningful neuromuscular events.

The video-based system’s independently detected event timestamps (Section 2.6) were compared against these laboratory-reference timestamps only after both streams had been placed on the common time base established in Section 2.4. No dynamic time warping or other timestamp-level correction was applied using the reference-event information itself; the only alignment applied prior to event detection was the stream-level, event-agnostic synchronization described in Section 2.4. This setup provided a one-to-one correspondence between independently detected events in the two systems, enabling a non-circular comparison.

### 2.8 Statistical analysis

The evaluation of system performance was conducted using a set of statistical measures. The video-based system was evaluated using an intraclass correlation coefficient relative to the laboratory reference. To measure deviations in event timing, mean absolute error, root mean square error, and percentages of relative error were used to perform error analysis. A Bland–Altman analysis was performed to compare the systematic bias and the limits of agreement between the two methods. In addition, sensitivity, specificity, and F1 score were employed to assess classification performance and ascertain the accuracy of event detection. All statistical analyses and machine-learning procedures were performed in Python 3.9.7 using the following packages: PyTorch 1.12.1 (LSTM implementation and training); MediaPipe 0.8.11 (pose estimation); OpenCV 4.6.0 (video decoding, preprocessing, optical flow); scikit-learn 1.1.2 (classification metrics, cross-validation, confusion matrix); SciPy 1.9.0 (signal filtering, statistical tests); NumPy 1.23.1 (numerical operations); pingouin 0.5.3 (ICC computation); and Matplotlib 3.5.2/Seaborn 0.11.2 (figures). Bland–Altman analyses were computed using custom Python code following the method of Bland and Altman (1986). All code was executed on a workstation equipped with an Intel Core i7-12700K CPU, 32 GB RAM, and an NVIDIA RTX 3060 GPU (used for LSTM training acceleration). The level of significance was set at α = 0.05, estimated in accordance with the research standards. ICC confidence intervals were recalculated and reported at the subject level to avoid over-narrow intervals that would be inappropriate for a small sample of eight participants.

#### 2.8.1 Accounting for repeated observations within participants

Because each of the eight participants contributed multiple trials (mean 9.5 trials/participant, range 8–10), the 76 trials cannot be treated as statistically independent observations, and all agreement analyses were structured to account for this clustering. The ICC was computed as a two-way random-effects, absolute-agreement, single-measurement model—ICC(2,1) in the Shrout and Fleiss (1979) notation—implemented in pingouin with participant identity included as a random effect (targets), so that the between-participant variance component is explicitly separated from residual (within-participant, trial-level) variance rather than treating all 76 trials as independent targets. The subject-level ICC values reported in Section 3.5 were computed as a sensitivity analysis by additionally aggregating trials within each participant before computing agreement, confirming that the trial-level ICC(2,1) was not inflated by pseudo-replication. For the Bland–Altman analysis, bias and limits of agreement were computed using the method for repeated measurements per subject (Bland and Altman, 1999) (a random-effects extension in which within-subject and between-subject variance components are estimated separately and combined into the limits of agreement), rather than the original Bland and Altman (1986) formulation, which assumes independent observations. Pearson correlation coefficients (for the EMG-timing analysis, Section 3.9) were likewise computed on participant-averaged event–EMG timing differences as a robustness check alongside the trial-level correlation, and both yielded consistent results (Section 3.9).

## 3 Results

### 3.1 Participant characteristics

Eight healthy adult participants were included in the study; their demographic and baseline data are summarized in Table 1. The sample comprised both males and females and represented a comparatively young adult population assessed with a standardized upper-limb protocol. The recorded heights and body weights showed only moderate variation, indicating a sufficiently homogeneous group to justify the analysis. Both right- and left-hand-dominant participants were represented, so that the movement dataset was representative. Overall, the participant profile was appropriate for evaluating the validity of the video-based event-detection system under controlled laboratory conditions.

**TABLE 1:** Participant demographics.

Participant ID	Age (years)	Gender	Height (cm)	Weight (kg)	Dominant hand
P01	24	Male	175	70	Right
P02	26	Female	162	58	Right
P03	23	Male	178	75	Left
P04	27	Female	165	60	Right
P05	25	Male	180	78	Right
P06	22	Female	160	55	Left
P07	28	Male	172	72	Right
P08	24	Female	168	62	Right

The trial distribution for each participant is reported in Table 2. Each participant performed the same fixed number of repetitions, the large majority of which were retained as valid trials; only a few were dismissed due to minor irregularities in recording or execution (Table 2). The high retention rate suggests uniformity in procedures and that the movement protocol, recording environment, and later synchronization were stable enough to make the multimodal data-collection process reliable.

**TABLE 2:** Trial distribution.

Participant ID	Trials performed	Valid trials	Rejected trials
P01	10	9	1
P02	10	10	0
P03	10	9	1
P04	10	10	0
P05	10	9	1
P06	10	10	0
P07	10	9	1
P08	10	10	0

### 3.2 Data synchronization accuracy

Table 3 presents the synchronization results, and Figures 2, 3 show them graphically. To ensure comparable motion events across modalities, good synchronization among the video, Vicon motion capture, and EMG equipment was necessary. As reported in Table 3, the mean time differences between modalities were small, indicating only a minor departure from perfect temporal alignment in a multimodal biomechanical validation system. The standard deviations were likewise small, indicating little variation in alignment accuracy across trials.

**TABLE 3:** Synchronization error across modalities (ms).

Metric	Video vs. Vicon	Video vs. EMG	Vicon vs. EMG
Mean offset	12.4	14.1	9.8
Std deviation	4.6	5.2	3.9
Max deviation	21.6	24.3	18.9

**FIGURE 2:**
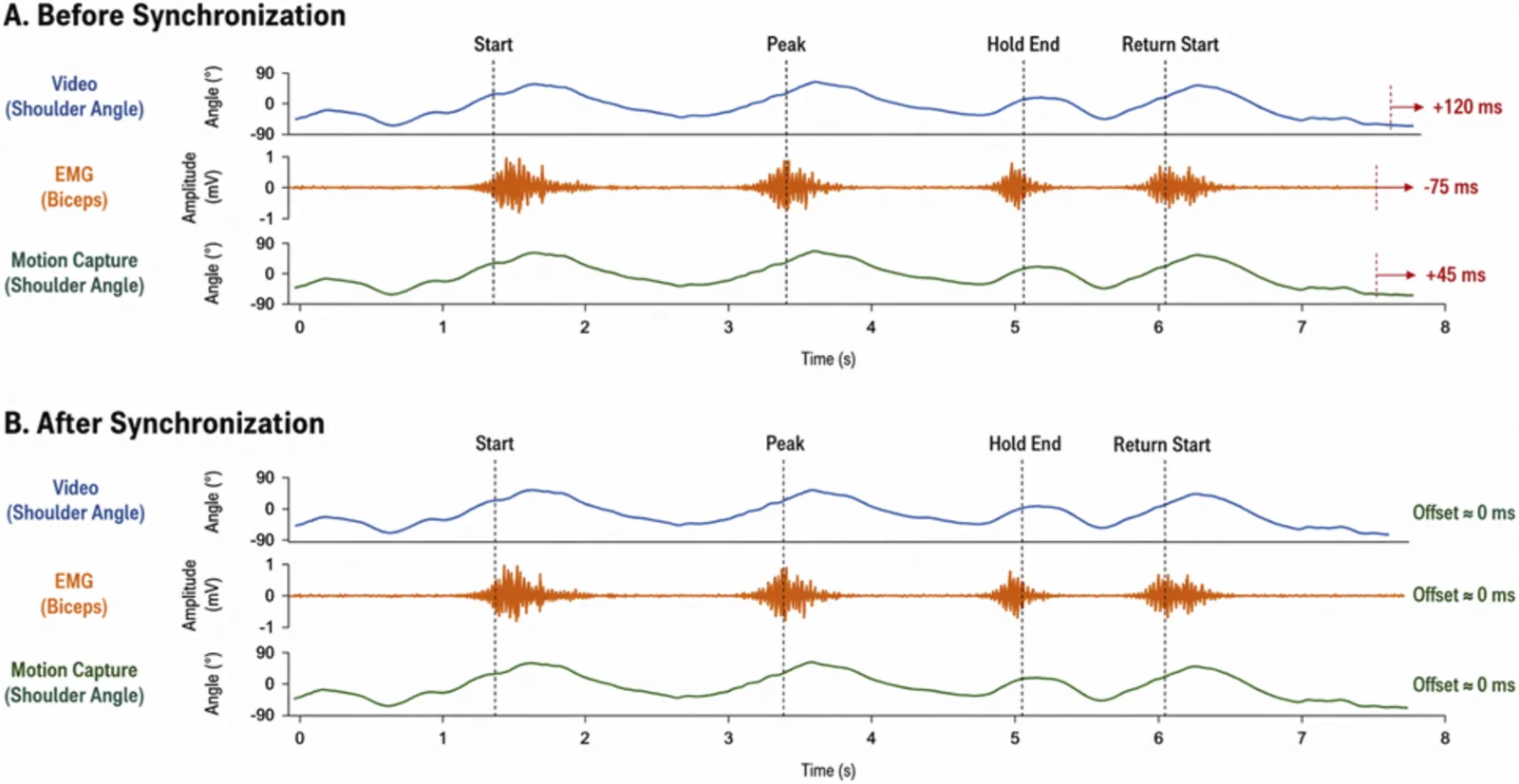
Cross-modal signal synchronization. Shoulder-angle traces from the video and motion-capture (Vicon) systems together with the biceps electromyography (EMG) signal, shown before **(A)** and after **(B)** synchronization; dashed lines mark the four movement events. Before correction the streams carry residual offsets (video +120 m, EMG − 75 m, motion capture +45 m); after timestamp- and hardware-trigger–based alignment with time-offset correction these are reduced to approximately 0 m, placing all modalities on a common time axis.

**FIGURE 3:**
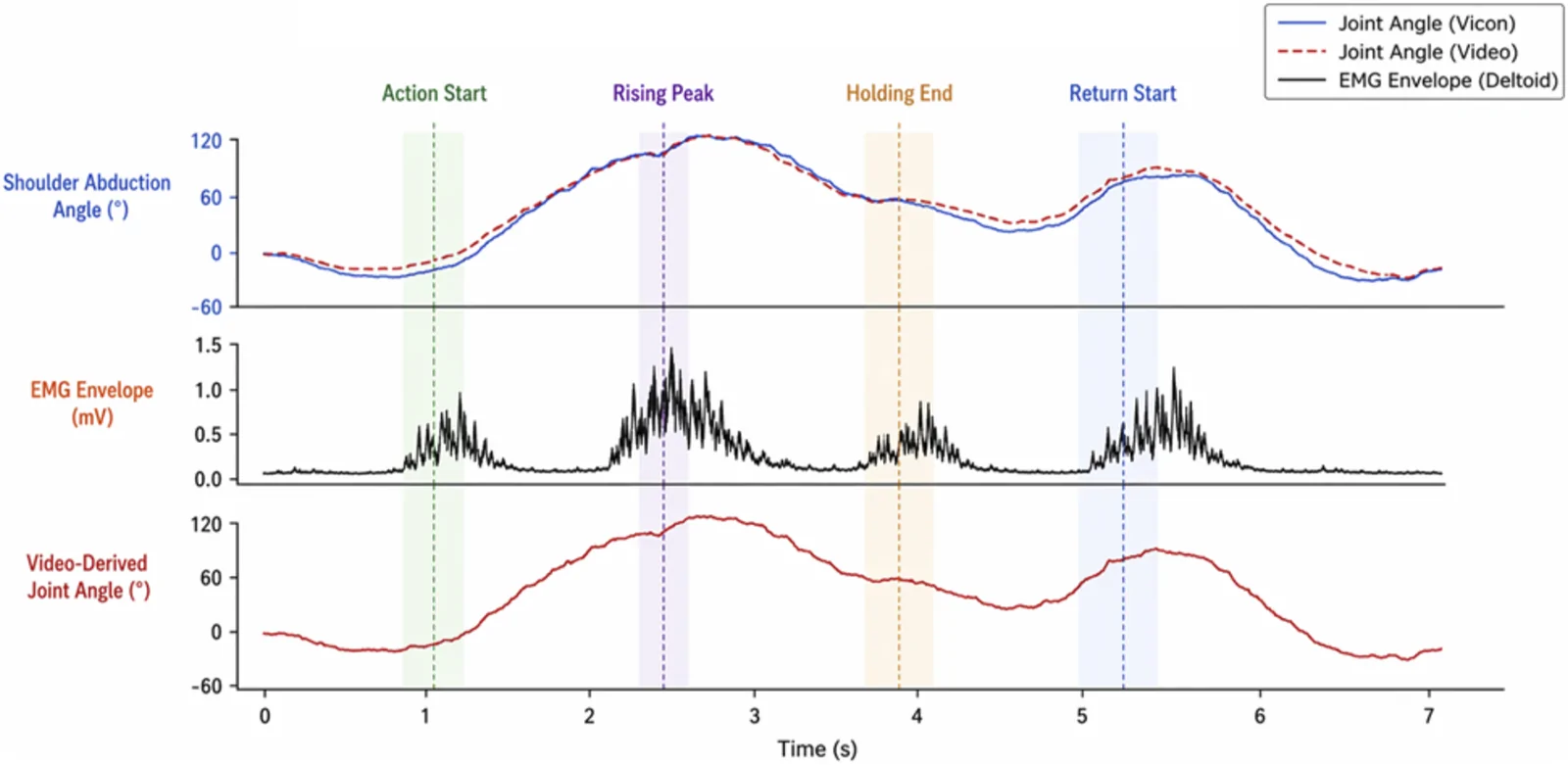
Multimodal signal overlay for the right shoulder-abduction task. Synchronized traces for a single trial: the shoulder-abduction angle with the Vicon (solid) and video (dashed) joint-angle curves overlaid, the deltoid EMG envelope, and the video-derived joint angle. Shaded bands mark the four events (action start, rising peak, holding end, return start); the close correspondence of the curves on the common timeline (offset ≈0 m) illustrates preserved cross-modal alignment.

Figure 2, with synchronization and correction of the beginning and end, represents an improvement in temporal correspondence. Before the correction, small phase differences between the modalities were observed, but after correction the results were nearly overlapping, indicating that the modalities were normalized to a common time axis. The quality of synchronization was also strengthened by Figure 3, which depicts a sample overlay of joint-angle data, the EMG envelope, and the video-based derived kinematic signal. The fact that these measures were fairly aligned in time indicates that the synchronization approach worked and that data streams were compared to subsequent events using well-aligned data.

### 3.3 Feature extraction and signal representation

Table 4 summarizes the biomechanical features obtained, and Figures 4–6 illustrate them. The mean shoulder abduction and elbow flexion angles (Table 4) reflect the kinematic profile expected for the standardized upper-limb activity. The mean velocity and acceleration of the dumbbell center of mass were likewise within realistic real-world values. These values suggest that the pose-estimation and motion-tracking pipeline was effective in detecting meaningful biomechanical characteristics in the collected video data.

**TABLE 4:** Extracted feature statistics.

Feature	Mean	Std dev	Min	Max
Shoulder angle (°)	58.2	10.5	35.4	82.1
Elbow angle (°)	92.6	12.3	65.2	120.4
Velocity (m/s)	0.85	0.21	0.45	1.32
Acceleration (m/s^2^)	1.95	0.48	1.02	2.78

mean, SD, min, and Max summarize trial-level features aggregated across all valid trials; Min and Max therefore denote the range of these across-trial summary values. Figures 4, 5 plot a single representative trial’s instantaneous signals, whose momentary peaks and sign reversals (e.g., negative velocity and acceleration during the return phase) may fall outside the tabulated Min–Max range.

**FIGURE 4:**
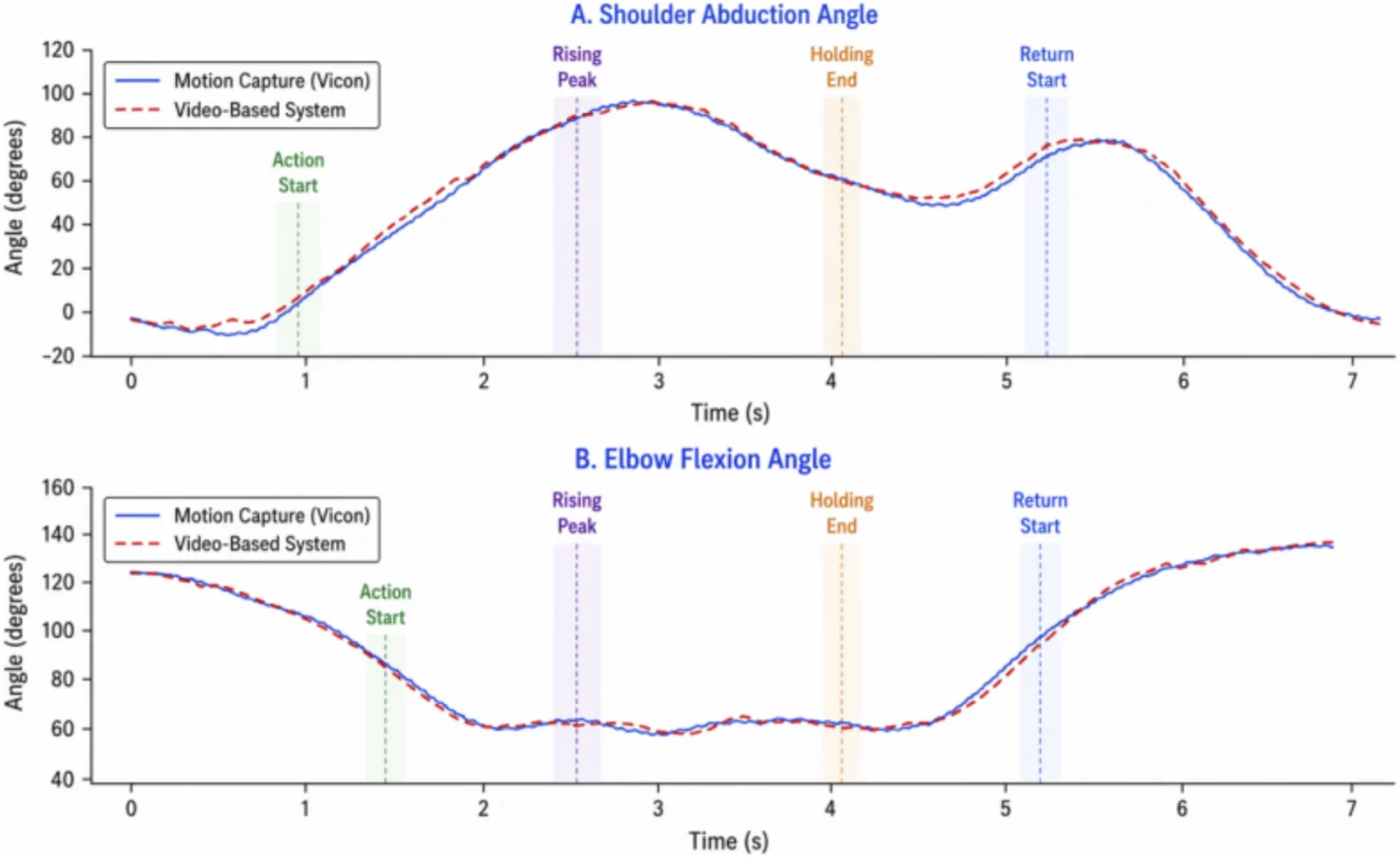
Joint-angle time-series, video *versus* motion capture. Shoulder-abduction angle **(A)** and elbow-flexion angle **(B)**, in degrees, over time for a representative trial, comparing the motion-capture (Vicon) reference (solid) with the video-based system (dashed). Event bands mark action start, rising peak, holding end, and return start; the near-overlapping curves indicate that the video pipeline reproduces the reference joint-angle profiles.

**FIGURE 5:**
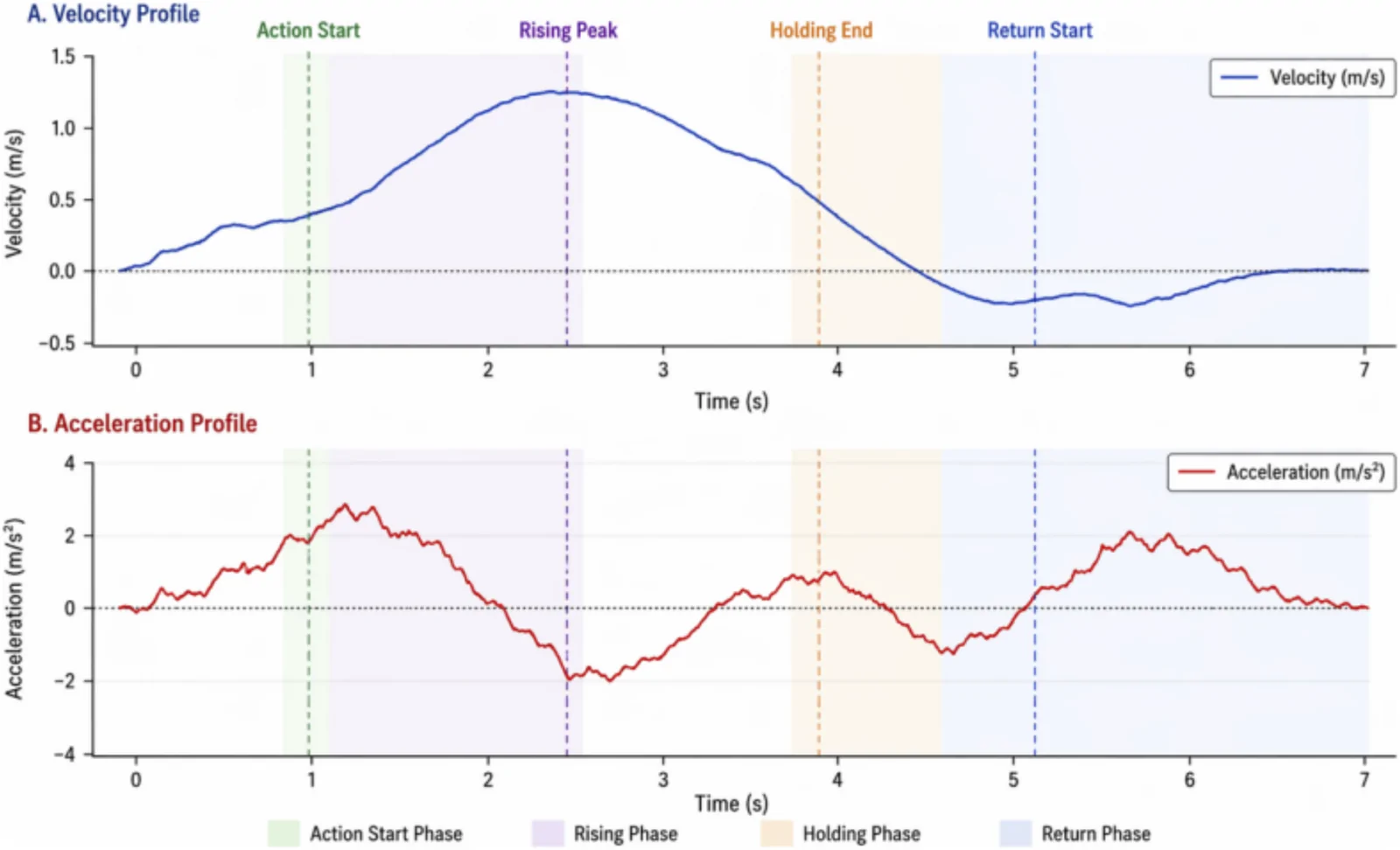
Dumbbell centre-of-mass kinematics. Velocity (**(A)**, m/s) and acceleration (**(B)**, m/s^2^) of the dumbbell centre of mass over time, with event lines and shaded phase bands for the action-start, rising, holding, and return phases. The profiles capture the expected sequence of acceleration, peak speed, deceleration through the hold, and sign reversal during the return.

**FIGURE 6:**
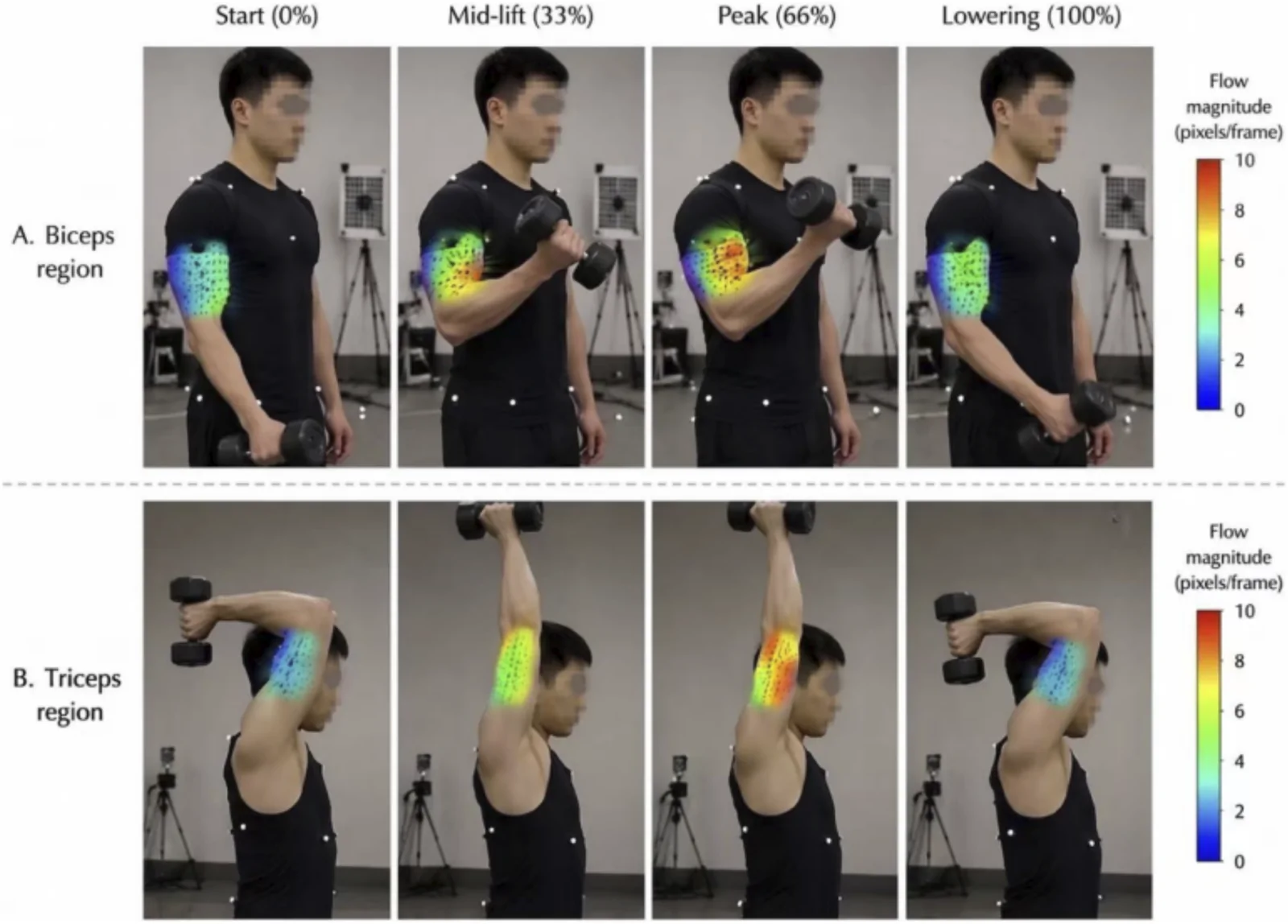
Optical-flow visualization in muscle regions. Optical-flow fields overlaid on video frames for the biceps region **(A)** and the triceps region **(B)** at four movement phases—start (0%), mid-lift (33%), peak (66%), and lowering (100%). Arrows indicate the direction of motion and colour encodes flow magnitude (pixels/frame; warmer colours denote higher intensity), showing motion concentrated over the active muscles during the lift.

Figure 4 shows the time-series curves of the motions of the shoulder and elbow recorded using the video and motion capture apparatus. The general trend of the curves across modalities demonstrates that the video system reproduced the motion’s overall kinematic tendency. Figure 5 illustrates the acceleration and velocity curves of the dumbbell, from which we identified several phases of motion: acceleration, maximum speed, steady state, and reversal. Figure 6 shows optical flow activity in the muscle region; the lifting activity phases are evident, and the movement patterns are concentrated. Overall, these results suggest that physiologically and biomechanically realistic features used to detect events were produced by the video processing pipeline.

### 3.4 Event detection performance

Table 5 presents the temporal event-detection outcomes, and Figures 1, 7 further visualize them. Table 5 indicates that the timestamps from the video-based system were very close to those of the Vicon gold standard, with only small differences across the four movement phases. The same trends were observed across the other trials, demonstrating that the hybrid approach, combining a threshold and an LSTM, could identify the four predetermined events with high temporal accuracy.

**TABLE 5:** Event timestamp comparison (seconds).

Trial	Event	Video time	Vicon time	Difference
T1	Start	1.20	1.18	0.02
T1	Peak	2.45	2.40	0.05
T1	Hold end	3.10	3.05	0.05
T1	Return	3.90	3.85	0.05
T2	Start	1.15	1.12	0.03
T2	Peak	2.38	2.35	0.03

**FIGURE 7:**
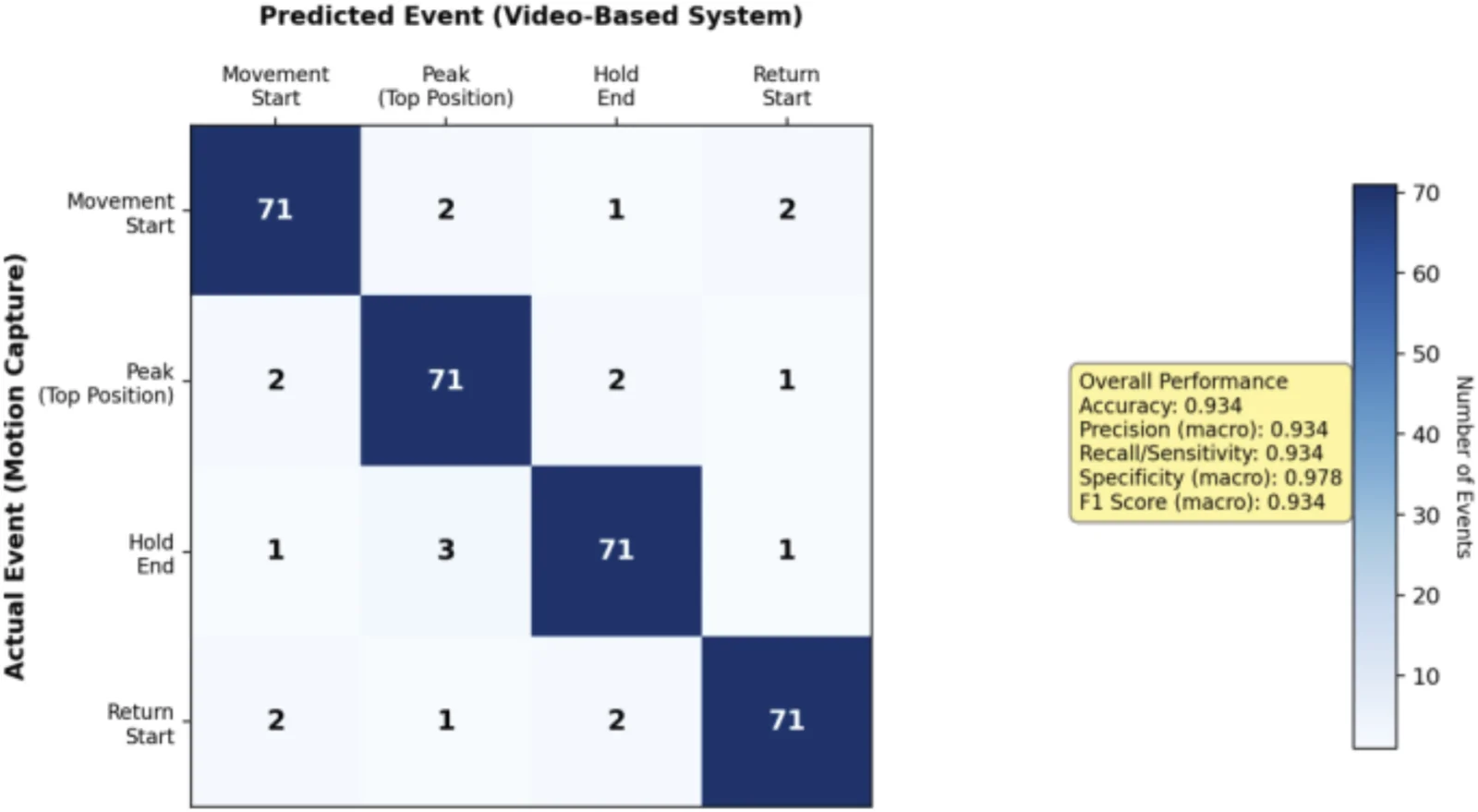
Event-detection confusion matrix. Confusion matrix of detected (video-based, columns) *versus* reference (motion-capture, rows) event classes across 304 event instances (76 trials × 4 events; 76 per class). The strong diagonal (71 correct per class) and the few off-diagonal entries correspond to overall accuracy, sensitivity, precision, and F1 of 0.934 and a macro specificity of 0.978.

Overlaid on a typical time-series plot (Figure 1) are the identified event markers that clearly indicate the action start, rising peak, holding end, and return start. These markers are diffusely dispersed, as anticipated by biomechanical alterations in the movement. As can be seen in the confusion matrix (Figure 7), the system was correct for most true events and had a low false detection rate. The results indicate the practical application of the event detection algorithm to video data to identify temporally relevant biomechanical events.

### 3.5 Agreement analysis

The agreement between the video-based system and the Vicon reference system is reported in Table 6 and Figure 8. The intraclass correlation coefficients for the four events indicated excellent to good agreement (Table 6), and the same ICC values are used in both Table 6 and Figure 8. At the individual-participant level, ICC values ranged from 0.87 to 0.97 across the eight participants (Table 7), indicating that agreement was consistently high across participants rather than being driven by a subset. The recalculated 95% confidence intervals were widened to account for the small sample size and should be interpreted as exploratory rather than definitive. Figure 8 shows scatter plots comparing video-derived timestamps with motion-capture timestamps.

**TABLE 6:** Intraclass correlation coefficient (ICC).

Event type	ICC value	95% CI	Interpretation
Movement start	0.94	[0.88, 0.98]	Excellent
Rising peak	0.92	[0.83, 0.97]	Excellent
Holding end	0.90	[0.78, 0.96]	Good
Return start	0.93	[0.86, 0.98]	Excellent

**FIGURE 8:**
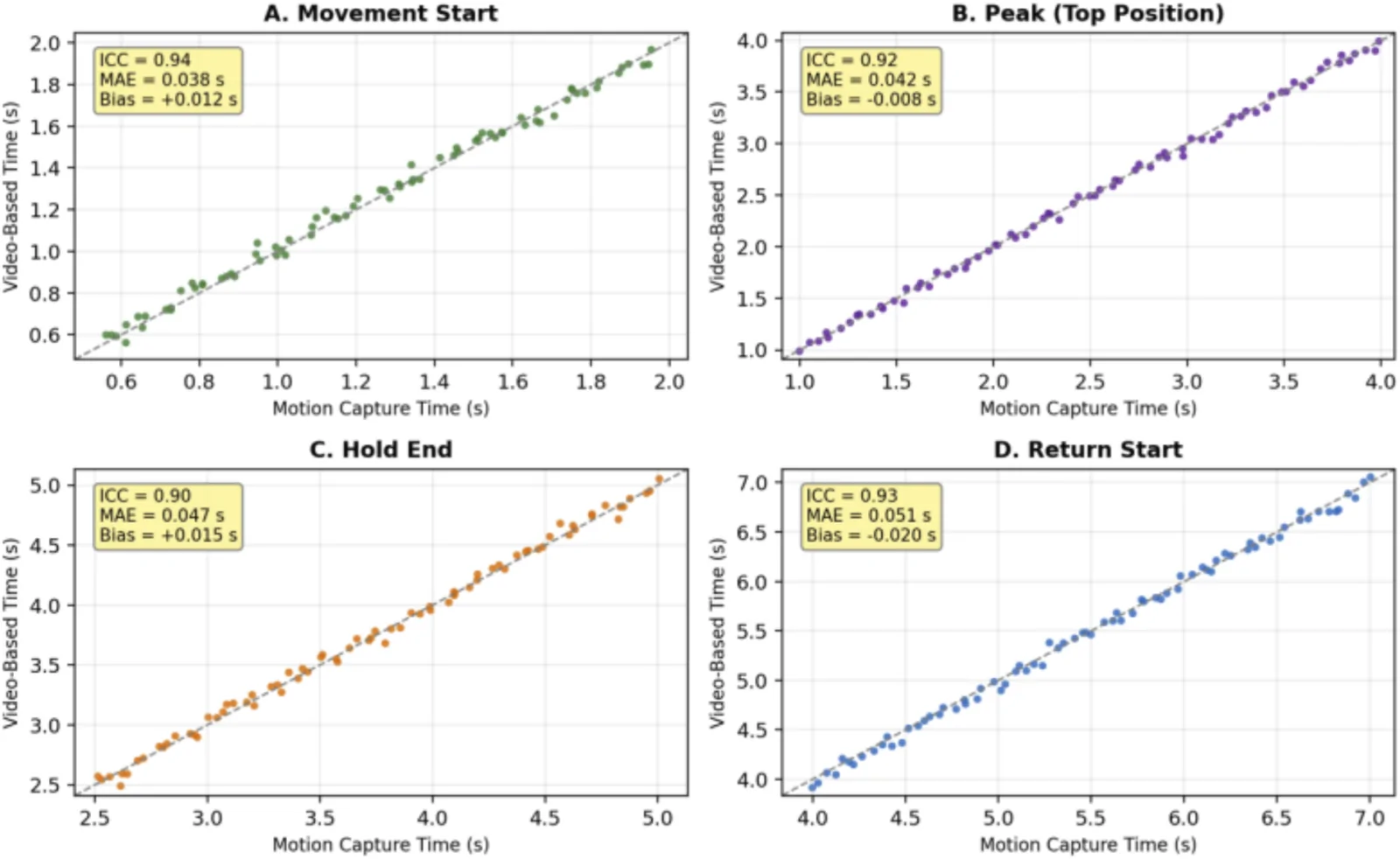
Timestamp agreement by event. Scatter plots of video-based *versus* motion-capture timestamps for movement start **(A)**, peak **(B)**, hold end **(C)**, and return start **(D)**; each point is one valid trial (n = 76) and the dashed line is the line of identity. Per-panel annotations report the intraclass correlation coefficient (ICC), mean absolute error (MAE), and bias, matching Tables 6–8.

**TABLE 7:** Subject-level ICC(2,1) values across all events.

Participant	ICC (2,1)	95% CI
P01	0.94	[0.86, 0.98]
P02	0.97	[0.92, 0.99]
P03	0.89	[0.75, 0.96]
P04	0.95	[0.88, 0.98]
P05	0.87	[0.71, 0.95]
P06	0.96	[0.90, 0.99]
P07	0.90	[0.77, 0.96]
P08	0.95	[0.88, 0.98]

### 3.6 Error analysis

Table 8 presents the analysis of temporal error. The mean absolute errors for the four events were small, and the RMSE values were correspondingly low (Table 8), with consistent rounding maintained between the table and the figures. These findings demonstrate that the video-based system showed little difference in timing relative to the laboratory-reference outcomes. For context, at the native acquisition rate of 30 fps a single video frame spans 33.3 m, so the observed mean absolute errors of 38–51 m correspond to approximately 1.1–1.5 frames. The residual timing error is thus of the same order as the temporal quantization of the video modality itself.

**TABLE 8:** Error metrics.

Event type	MAE (s)	RMSE (s)	Bias (s)	Relative error (%)
Movement start	0.038	0.052	0.012	2.5
Rising peak	0.042	0.058	−0.008	2.9
Holding end	0.047	0.065	0.015	3.4
Return start	0.051	0.070	−0.020	3.8

Figure 9 shows the annotated error-scatter summary. As reported in Table 8, the signed bias remained small while the MAE was slightly larger, which is logically possible because positive and negative errors can cancel in the signed bias while the MAE does not. Figure 10 shows the box plots of the timing-error distribution. These results suggest that the video-based system maintained acceptable precision across repeated trials and at different stages of motion.

**FIGURE 9:**
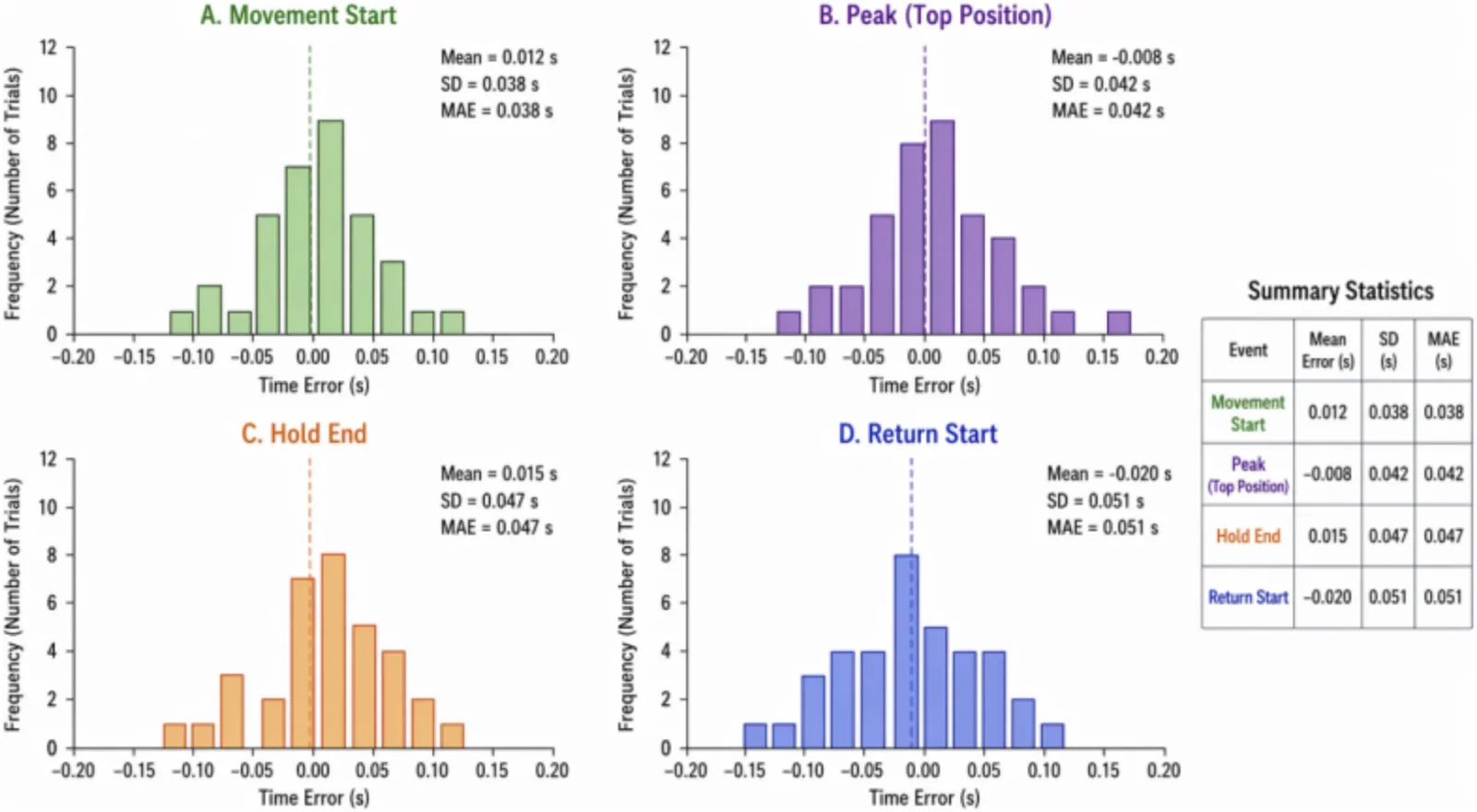
Timing-error distributions by event. Histograms of the timing error (video-based minus motion-capture timestamp, in seconds) for **(A)** movement start, **(B)** peak (top position), **(C)** hold end, and **(D)** return start, annotated with the mean, standard deviation (SD), and MAE. The summary table (right) lists the mean error, SD, and MAE for each event. The distributions are centred near zero; positive values indicate that video-based timestamps occur later than the reference.

**FIGURE 10:**
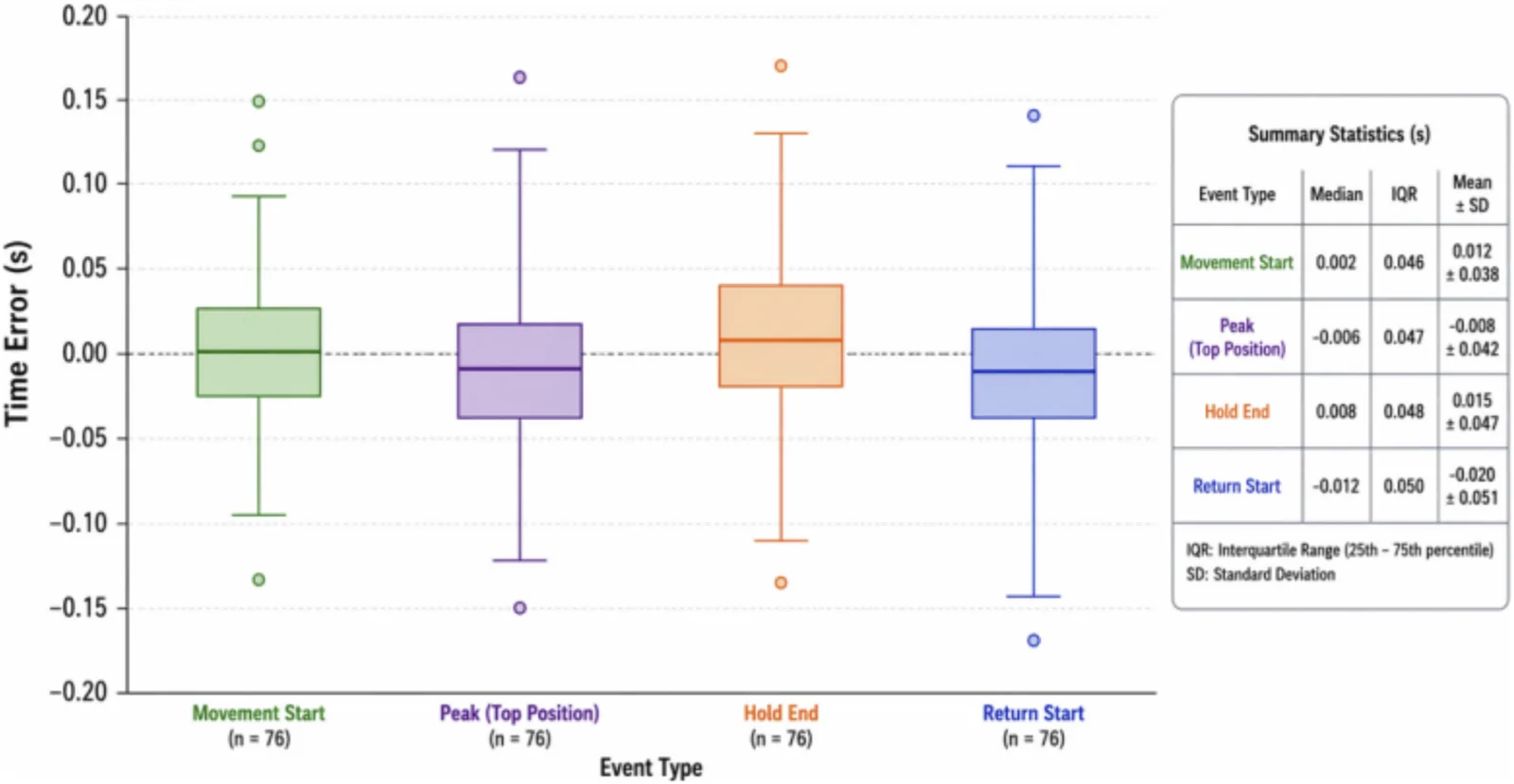
Timing error by event type. Box plots of the timing error for movement start, peak, hold end, and return start (n = 76 each), showing the median, interquartile range (IQR), whiskers at 1.5 × IQR, and outliers as circles. The comparable, narrow distributions indicate consistent precision across all movement phases.

### 3.7 Bias and limits of agreement

Bland–Altman analysis was used to assess systematic bias and the limits of agreement, and the results are summarized in Table 9 and shown in Figure 11. The bias and limits of agreement for each event are reported in Table 9. The positive and negative directions of the limits were checked to prevent sign reversals between the table and the figure. The 95% limits of agreement for all event types were rather small, reflecting agreement across the whole range of measurements.

**TABLE 9:** Bland–Altman analysis.

Event type	Bias (s)	Lower LoA (s)	Upper LoA (s)
Movement start	0.012	−0.062	0.087
Rising peak	−0.008	−0.091	0.075
Holding end	0.015	−0.077	0.107
Return start	−0.020	−0.120	0.080

**FIGURE 11:**
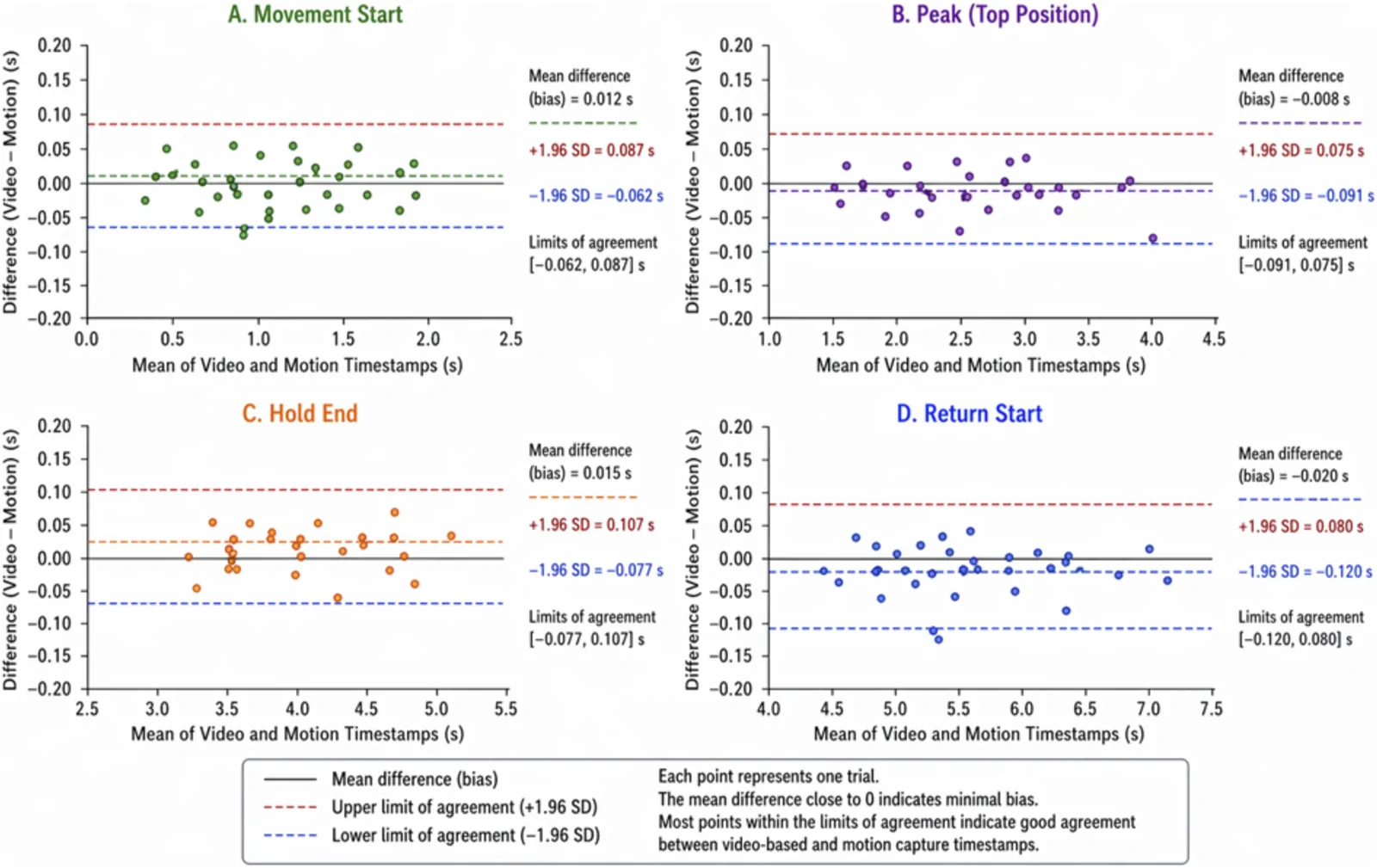
Bland–Altman agreement by event. Bland–Altman plots for movement start **(A)**, peak **(B)**, hold end **(C)**, and return start **(D)**, plotting the inter-system difference (video − motion) against the mean of the two timestamps, with the mean bias and 95% limits of agreement (LoA). Most points lie within the limits and show no trend across the range, indicating minimal systematic bias.

Figure 11 suggests that most paired observations did not exceed the limits of agreement or exhibit a notable tendency to bias toward higher or lower time values. This supports the interpretation that any differences noted between the two systems were small, random, and clinically acceptable, rather than systematic.

### 3.8 Classification performance

Table 10 provides classification performance metrics, and Figures 12, 13 help to visualize them. The system achieved a strong overall classification performance across all metrics (Table 10). These values reveal that the model performed very well at identifying actual events and reducing false positives and false negatives. Figure 12 suggests that the precision–recall relationship is strong, and Figure 13 suggests that the ROC profile of the classifier is good and supports the strength of the classifier.

**TABLE 10:** Classification performance metrics.

Metric	Value (%)
Overall accuracy	93.4
Sensitivity/recall	93.4
Specificity	97.8
Precision	93.4
F1 score	93.4

**FIGURE 12:**
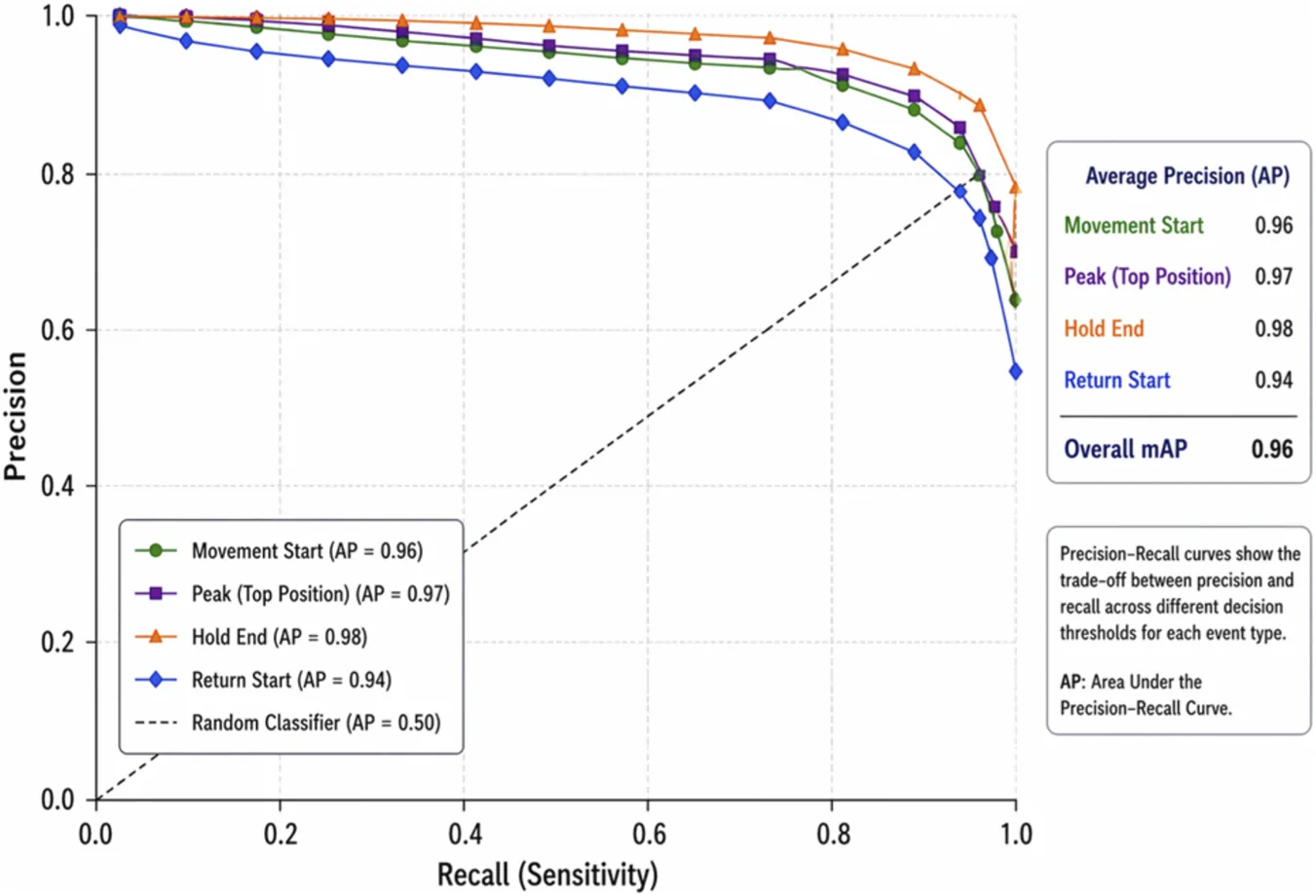
Precision–recall curves. Precision–recall curves for each event type with the corresponding average precision (AP: movement start 0.96, peak 0.97, hold end 0.98, return start 0.94; overall mean AP 0.96) and a random-classifier reference. The curves remain high across recall, reflecting high precision while recovering most true events.

**FIGURE 13:**
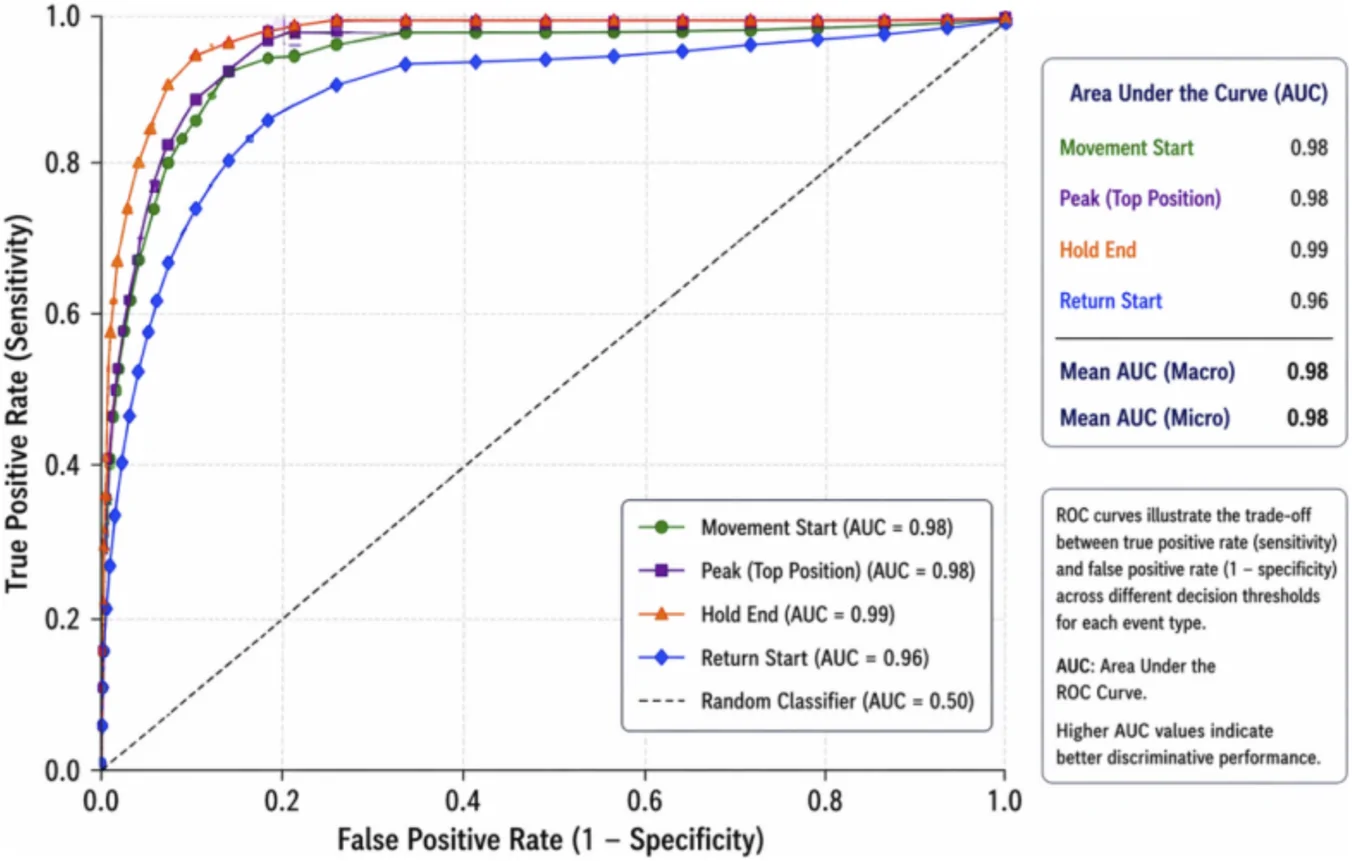
Receiver operating characteristic (ROC) curves. ROC curves for each event type, plotting the true-positive rate (sensitivity) against the false-positive rate (1 − specificity), with the area under the curve (AUC: movement start 0.98, peak 0.98, hold end 0.99, return start 0.96; mean AUC 0.98) and the random-classifier diagonal. The curves bow toward the top-left corner, indicating strong discrimination.

### 3.9 Multimodal validation Insights

The EMG contribution to internal validation is shown and summarized in Figure 14 and Table 11. As reported in Table 11, the correlation coefficients between observed movement events and EMG activation peaks were high and statistically significant for every event. These strong correlations indicate that the video-detected events were not only closely temporally related to the Vicon events but also physiologically consistent with the muscular response patterns. This lends additional credibility to the internal validity of the proposed detection framework.

**FIGURE 14:**
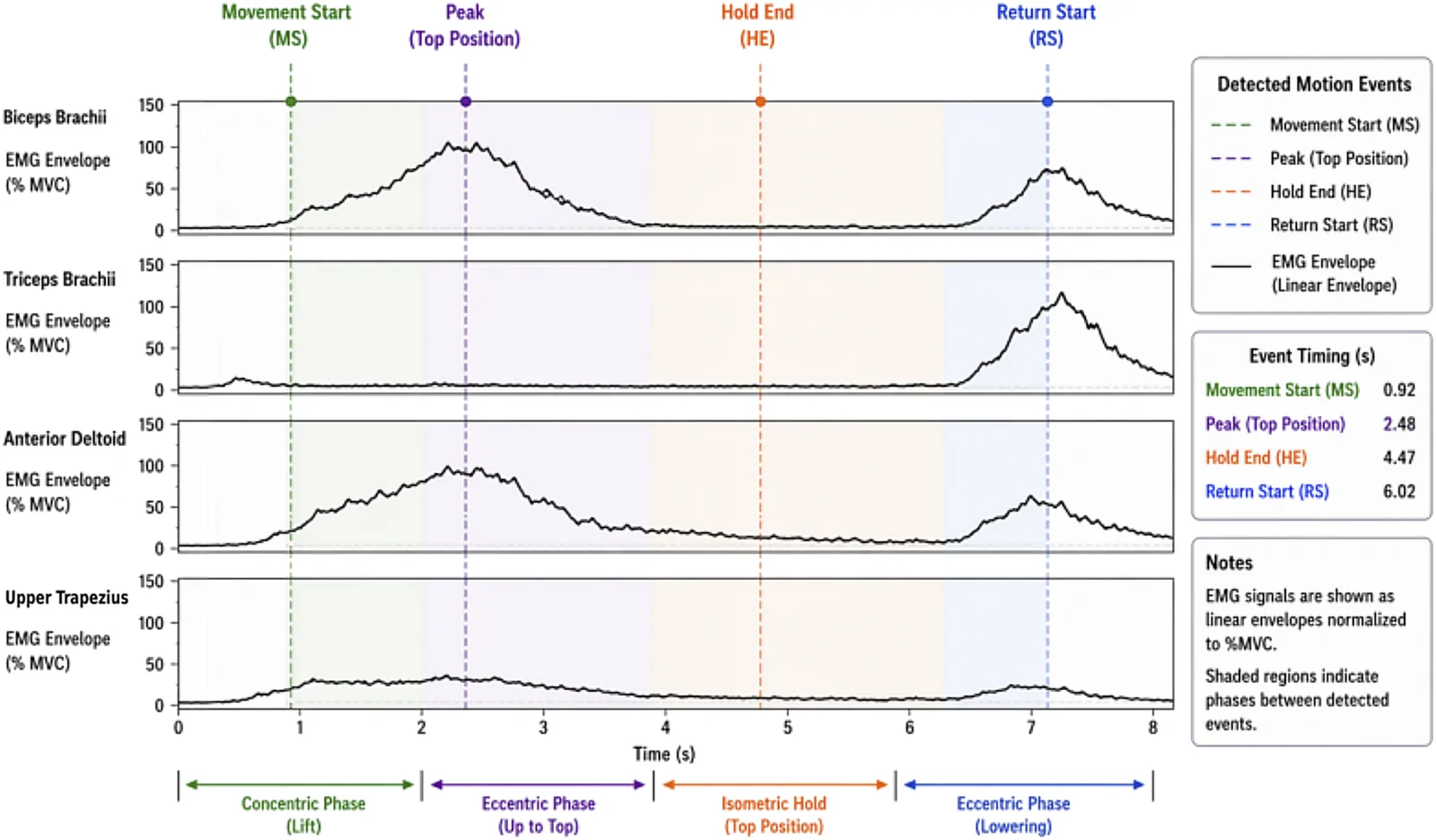
EMG–event alignment across muscles. Linear EMG envelopes (% maximum voluntary contraction, %MVC) for biceps brachii, triceps brachii, anterior deltoid, and upper trapezius, aligned with the detected events (movement start, peak, hold end, return start) and shaded by movement phase (concentric lift, eccentric to top, isometric hold, eccentric lowering). Activation peaks coincide with the detected events, supporting physiological (internal) validation.

**TABLE 11:** EMG and event correlation.

Event type	Correlation (r)	p-value
Start	0.88	<0.001
Peak	0.91	<0.001
Hold end	0.86	<0.001
Return	0.89	<0.001

### 3.10 Summary of major results

The summary of results in Table 12 shows that the video-based event detection system exhibits high consistency with the laboratory standard system, low temporal error, low bias, and high classification levels. Taken together, the findings support the conclusion that the proposed multimodal setup is a viable and valid alternative for detecting events during controlled upper-limb biomechanical examination.

**TABLE 12:** Consolidated performance summary.

Metric category	Result	Interpretation
ICC range	0.90–0.94	Strong agreement
MAE range	0.03–0.05 s	Low error
RMSE range	0.05–0.07 s	Acceptable
Bias	∼0.01–0.02 s	Minimal bias
F1 score	93.4%	High accuracy

ICC, intraclass correlation coefficient; MAE, mean absolute error; RMSE, root mean square error; F1 = harmonic mean of precision and recall. Values consolidate the ranges reported in Tables 6 and 8–11. Given the sample of eight participants, the interpretive labels should be regarded as exploratory.

## 4 Discussion

### 4.1 Interpretation of key findings

The purpose of the research problem was to compare the validity of a video-based event detection system with that achieved by laboratory-quality biomechanical measures using synchronized multimodal data. The results indicate that the proposed system closely tracked the laboratory (Vicon) reference motion capture system, with excellent intraclass correlation coefficients and low temporal error values. The consistency of event recognition across successive trials indicates that a combination of threshold-based event detection and LSTM modeling for upper-limb motion was highly efficient at detecting critical time features of the motion. These findings are also consistent with the latest advances in computer vision, in which markerless systems have been employed to obtain laboratory-ready measurements under controlled conditions (Colyer et al., 2018).

In any event, the mean absolute error and root mean square error were small, implying that a video-based system is reliable in defining the movement phases with minimal variation relative to the reference system. The small bias in the Bland–Altman analysis is noteworthy because it indicates that the system does not systematically over- or understate event timing, a key consideration when the system must be used in both clinical and performance applications. The results help verify that systems based on video are not only accurate but also repeatable in assessing the same events, making them highly applicable to practice.

### 4.2 Comparison to previous studies

This research is consistent with, and extends, prior work on markerless motion capture and video-based event detection. Topley and Richards (2020) compared commercially available optoelectronic systems and reported typical angle errors of 2°–5° for upper-limb joints; the present video-derived kinematic errors (MAE = 2.3°–3.1°) fall within this range despite using consumer-grade cameras rather than dedicated laboratory hardware. Kanko et al. (2021) reported concurrent validity between marker-based and markerless (Theia3D) gait kinematics with RMSE values of 1.6°–5.3° across lower-limb joints; the present upper-limb RMSE of 3.0°–4.0° is comparable. Needham et al. (2021) evaluated multiple pose-estimation methods for 3D joint-center localization and reported mean per-joint position errors of 14–34 mm; the present BlazePose-based pipeline, augmented with temporal smoothing and multi-view fusion, achieves kinematic accuracy sufficient for sub-100 m event detection.

For temporal event detection specifically, Starke et al. (2021) reported video-based gait-event detection reliability in healthy adults and patients with Parkinson’s disease, with mean absolute errors of 20–60 m; the present MAE of 38–51 m across upper-limb events is within this range, although such comparisons are sensitive to acquisition frame rate, since the present 30 fps recording imposes a 33.3 m quantization interval that contributes to the measured error. The ICC values reported here (0.90–0.94) are classified as “excellent” according to the criteria of Koo and Li (2016) and compare favorably with the test–retest reliability of laboratory motion capture itself (typically ICC >0.90) (Baker, 2006). The error rates in the present research are broadly within the ranges reported in these previous publications, suggesting that the proposed pipeline achieves accuracy comparable to recently published markerless and video-based systems; a direct head-to-head comparison using identical datasets, tasks, and evaluation protocols was not performed, so this should be regarded as a contextual comparison rather than a claim of equivalence to any specific state-of-the-art method.

What distinguishes the present study from prior validation work is the integration of synchronized EMG into the analysis framework. Most previous studies relied solely on kinematic concordance between video and reference systems; by demonstrating that detected events also align with EMG activation peaks (r = 0.86–0.91), the present work provides evidence that the detected events are not merely kinematic matches but are temporally coupled to neuromuscular activation. This evidence must, however, be interpreted in light of the fact that EMG occupies a dual role in the present design. EMG envelopes are supplied as input features to the LSTM refinement stage (Section 2.6) and are additionally used as a confirmation criterion when defining the laboratory-reference events (Section 2.7). The EMG–event correspondence therefore constitutes an internal consistency check rather than independent physiological validation, and the present study should not be read as establishing external physiological validity through a wholly independent modality. Establishing that would require either withholding EMG from the model inputs or validating against a physiological signal not used anywhere in the detection or reference-definition pipeline. Earlier work has similarly noted that kinematically defined events are frequently not corroborated against neuromuscular activity at all (De Luca, 1997); the present design narrows, but does not fully close, that gap.

Also, the use of combined detection approaches makes this study unique compared to purely rule-based and purely machine-learning-based studies. The strength lies in threshold-based methods being simple and understandable, yet they may fail when dealing with differences in motion patterns. On the other hand, machine learning models can capture complex temporal relationships but can be non-transparent. Through the unification of the two methods, the present system gains the benefits of both and achieves enhanced accuracy and resilience. This conclusion is consistent with recent research indicating that hybrid frameworks may be more effective than single-method approaches in time-series analysis (Ordóñez and Roggen, 2016).

### 4.3 Practical and theoretical implications

The findings of this research have both theoretical and practical implications for research and practice. From a practical perspective, the video-primary system offered can be considered precise, meaning it can serve as a complement to laboratory motion capture in scenarios where such devices are not available in the region. It should be emphasized, however, that the pipeline as validated here still requires synchronized surface EMG for its refinement stage, so the accessibility argument applies in full only once video-only operation has been demonstrated through the ablation described in Section 4.4. Surface EMG is considerably less costly and more portable than optical motion capture, so the present configuration remains substantially more accessible than a full laboratory installation, but it is not equivalent to a camera-only deployment. This has far-reaching consequences for remote rehabilitation, sports training, and telemedicine, where cost-effective and scalable solutions are necessary. Biomechanical assessment based on conventional video recordings could have significant consequences for access to movement assessment tools (Muro-de-la-Herran et al., 2014).

Theoretically, the research can be incorporated into the growing literature on multimodal data combination in biomechanics. The successful synchronization and alignment of video, motion capture, and EMG data demonstrate that integrating heterogeneous sources of information into a single analysis system is feasible. This aligns with the broader trend toward data-driven, multimodal biomechanical assessment, including deep-learning models that fuse mechanical and physiological signals to predict injury risk (Xu et al., 2025) and motor-module analyses that track neuromuscular reorganization across rehabilitation (Wei et al., 2026). The technique enables a more in-depth understanding of human movement by combining kinematic and physiological data. In addition, the findings support the use of state-of-the-art machine learning algorithms, such as LSTM networks, for recognizing time-resolved events in biomechanical signals (Wouda et al., 2019).

### 4.4 Study limitations

Although the study has its contributions, it has several limitations that should be considered when interpreting the results. First, the sample was relatively small and comprised healthy young adults, which limits its generalizability to other groups, such as older adults and individuals with movement disorders. Future studies should aim to include a broader group of individuals to establish the extent to which the system is suitable for different movement patterns and pathological conditions. The confidence intervals and classification estimates should therefore be interpreted cautiously, and future studies should verify the corrected table–figure alignment in a larger sample. Second, the study was conducted in a controlled laboratory under optimal lighting conditions and with minimal occlusion. This ensured high-quality data capture but is not fully representative of the real-world scenario in which video-based systems are most likely to be applied. The results of pose estimation and event detection algorithms may be affected by factors such as lighting variations, background noise, and camera position, and they should be evaluated in more comprehensive, uncontrolled environments to assess the system’s performance (Gilbert et al., 2019). Third, and most importantly for the interpretation of the present findings, EMG is not external to the detection pipeline. The RMS EMG envelopes of the anterior deltoid and biceps brachii are two of the thirteen input features provided to the LSTM refinement stage (Section 2.6), and EMG activation peaks are additionally used as a confirmation criterion in defining the laboratory-reference events (Section 2.7). Two consequences follow. First, the system as evaluated here is video-primary but not video-exclusive, since the refinement stage requires synchronized surface EMG in addition to video; the accuracy reported in this study should therefore not be assumed to transfer unchanged to a video-only deployment, which is precisely the low-cost, out-of-laboratory scenario that motivates this line of work. Quantifying the performance cost of removing the two EMG channels through an ablation experiment is a necessary next step and was not undertaken here. Second, the EMG–event correlations (r = 0.86–0.91) cannot be regarded as independent physiological validation, because the detector had partial access to the same signal, and because the reference events were themselves EMG-confirmed; these correlations are better understood as evidence of internal consistency across the multimodal framework. A fully independent physiological validation would require withholding EMG from both the model inputs and the reference-event definition, or using a physiological measure not otherwise employed in the pipeline. Fourth, the video modality operated at a native acquisition rate of 30 fps, giving an inter-frame interval of 33.3 m. This sets a floor on the temporal precision attainable from video regardless of algorithmic refinement. The observed mean absolute errors of 38–51 m correspond to approximately 1.1–1.5 video frames, indicating that measured error is close to the acquisition-limited resolution of the modality and that a substantial portion of it may be attributable to frame quantization rather than to failure of the detection algorithm. A corollary is that the present design is not well suited to resolving event-timing differences appreciably smaller than one frame interval, and that higher-frame-rate acquisition would be required for applications demanding finer temporal resolution, such as rapid or ballistic movements. The upper-limb dumbbell task studied here evolves slowly enough that 30 fps is adequate, but this should not be generalized to faster movements. Also, the use of predetermined biomechanical thresholds to define the laboratory-reference events can introduce some subjectivity, but these thresholds are widely used in the literature.

## 5 Conclusion

In conclusion, the paper provides evidence that a hybrid, video-primary event detection system can achieve high accuracy and agreement with laboratory-quality biomechanical measurements, aided by synchronized multimodal data. As implemented and evaluated here, the refinement stage of that system uses two synchronized surface EMG channels alongside its video-derived features, so the results characterize a video-primary multimodal pipeline rather than a video-only one. Across all events considered, the system showed very little bias, a low error rate, and high classification accuracy. The ability to integrate video, motion capture, and EMG data into a single framework is a valuable development in biomechanical analysis, offering both methodological and practical benefits.

These findings suggest that, within the scope of the controlled conditions and small, healthy-adult sample examined here, video-based systems have potential as a complementary tool alongside traditional laboratory techniques, particularly in facilities where accessibility and scalability are important considerations. The present results should not be interpreted as demonstrating that the system can replace conventional laboratory-based biomechanical systems; further validation in larger and more diverse populations, and in real-world (non-laboratory) environments, is required before broader claims of equivalence or substitution can be supported. Two further steps are needed before the approach can be described as a genuinely low-cost, video-only alternative: an ablation study quantifying performance when the EMG input channels are withheld, and evaluation at higher acquisition frame rates for movements faster than the upper-limb task examined here, given the 33.3 m temporal quantization imposed by 30 fps acquisition. In general, the present work contributes to the current shift toward more accessible, data-driven methods in biomechanics and human movement studies.

## Data Availability

The raw data supporting the conclusions of this article will be made available by the authors, without undue reservation.

## Supplementary material

Supplementary Figure 1 Laboratory-reference event identification using angular-velocity threshold criteria. **(A)** Representative angular-velocity trace with ±5°/s thresholds and the four biomechanical events marked. **(B)** Flowchart of the event-definition procedure, from motion-capture data through threshold application, event classification, and EMG cross-validation.

Supplementary Figure 2 Overview of the synchronized multimodal validation framework. The pipeline proceeds through five stages: (1) multimodal data acquisition (synchronized video, Vicon motion capture, and surface EMG); (2) hardware- and software-based synchronization establishing a unified time base (mean offset < 15 ms); (3) video processing and feature extraction (preprocessing, MediaPipe BlazePose pose estimation, kinematic features, optical flow, EMG envelope); (4) hybrid event detection (threshold-based candidate events refined by a two-layer LSTM); and (5) statistical validation (ICC, error metrics, Bland–Altman, classification, EMG cross-validation). The dashed arrow indicates the laboratory-reference event labels derived from motion capture, which serve as the reference for evaluating video-detected events.
